# Tocotrienols: Exciting Biological and Pharmacological Properties of Tocotrienols and other Naturally Occurring Compounds, Part I

**Published:** 2022-05-12

**Authors:** AA Qureshi

**Affiliations:** Department of Biomedical Science, School of Medicine, University of Missouri-Kansas City, USA

**Keywords:** Tocotrienols, α-tocopherol, Resveratrol, Vitamin D3, Quercetin, Dexamethasone, HEK293T cells, Chickens, Mice, Dog, Humans, Lipid metabolism, Inflammatory biomarkers

## Abstract

Inflammation has been implicated in cardiovascular disease and tocotrienols are potent hypocholesterolemic agents that reduce β-hydroxy-β-methyl-glutaryl coenzyme A reductase activity, which is degraded *via* the ubiquitin-proteasome pathway. Impact of various tocotrienols (α-, γ-, or δ-tocotrienol) treatments inhibit the chymotrypsin-like activity of 20S rabbit muscle proteasome (>50%) in RAW 264.7 cells and BALB/c mice. Moreover, the effect of various tocotrienols (α-, γ-, or δ-tocotrienol), α-tocopherol, quercetin, riboflavin, (−) Corey lactone, amiloride, dexamethasone supplemented diets fed to chickens (4-weeks) resulted in reduction of total cholesterol, LDL-cholesterol, and triglycerides. This trend was also observed in macrophages from RAW 264.7 cells, in LPS-induced thioglycolate-elicited peritoneal macrophages derived from C57BL/6, BALB/c, LMP7/MECL-1^−/−^, and PPAR-α^−/−^ knockout mice from young (4-week-old) and senescent (42-week-old) mice, resulting in significant inhibition of TNF-α and nitric oxide levels (30% to 70%), blocked degradation of P-IκB protein, and decreased activation of NF-κB, followed gene suppression of mRNA levels of TNF-α, IL-1β, IL-6, and *iNOS*. In human study, normal or hypercholesterolemic subjects administered two capsules/d of NS-7 or NS-6 (4-weeks) showed decrease in serum CRP, NO, γ-GT, total cholesterol, LDL-cholesterol, and triglycerides levels in normal as compared to hypercholesterolemic subjects (12% to 39%). In second study, hypercholesterolemic subjects were given increasing doses of δ-tocotrienol (125 mg, 250 mg, 500 mg, and 750 mg/day) plus AHA Step-1 diet (4-weeks). The most effective dose of tocotrienols (250 mg/day) may be used to lower serum NO (40%), CRP (40%), MDA (34%), γ-GT (22 %), and inflammatory cytokines IL-1α, IL-12, IFN-γ by 15% to 17%, and increase TAS levels by 22%.

## Introduction

My journey of studying tocotrienols started thirty years ago, when I first reported the isolation and biological function of α-tocotrienol as a hypocholesterolemia agent from barley in 1986 [1]. This was acknowledged by late Byron J Richards in his article of “Tocotrienols: Twenty Years of Dazzling Cardiovascular and Cancer Research in 2014, the first recognition of tocotrienols as a regulator of cholesterol occurred in a 1986 study in which α-tocotrienol was isolated from barley and fed to chickens. α-Tocotrienol reduced the rate of synthesis of cholesterol by the liver, in turn reducing total cholesterol and LDL-cholesterol. In April 1991, the American Journal of Clinical Nutrition published an animal and two human studies, triggering an explosion in scientific interest in tocotrienols, which continues to this day [2–5]”. This fact was further supported earlier by Dr. Barry Tan in his review article in Whole Foods (2012) of “Tocotrienols: Emerging Science and innovation of vitamin E Part 4: A Review of Groundbreaking Tocotrienol Research [6]”, and Tan et al. have published two books under the title “Tocotrienols vitamin E beyond tocopherols”, in which they have reported most of outstanding results of several world investigators on the biological properties of tocotrienols [7]. Mr. late Byron J Richards has summarized some of the results on tocotrienols published from my laboratory during 1990–2010 in his above-mentioned article [2].

**Figure 1:**
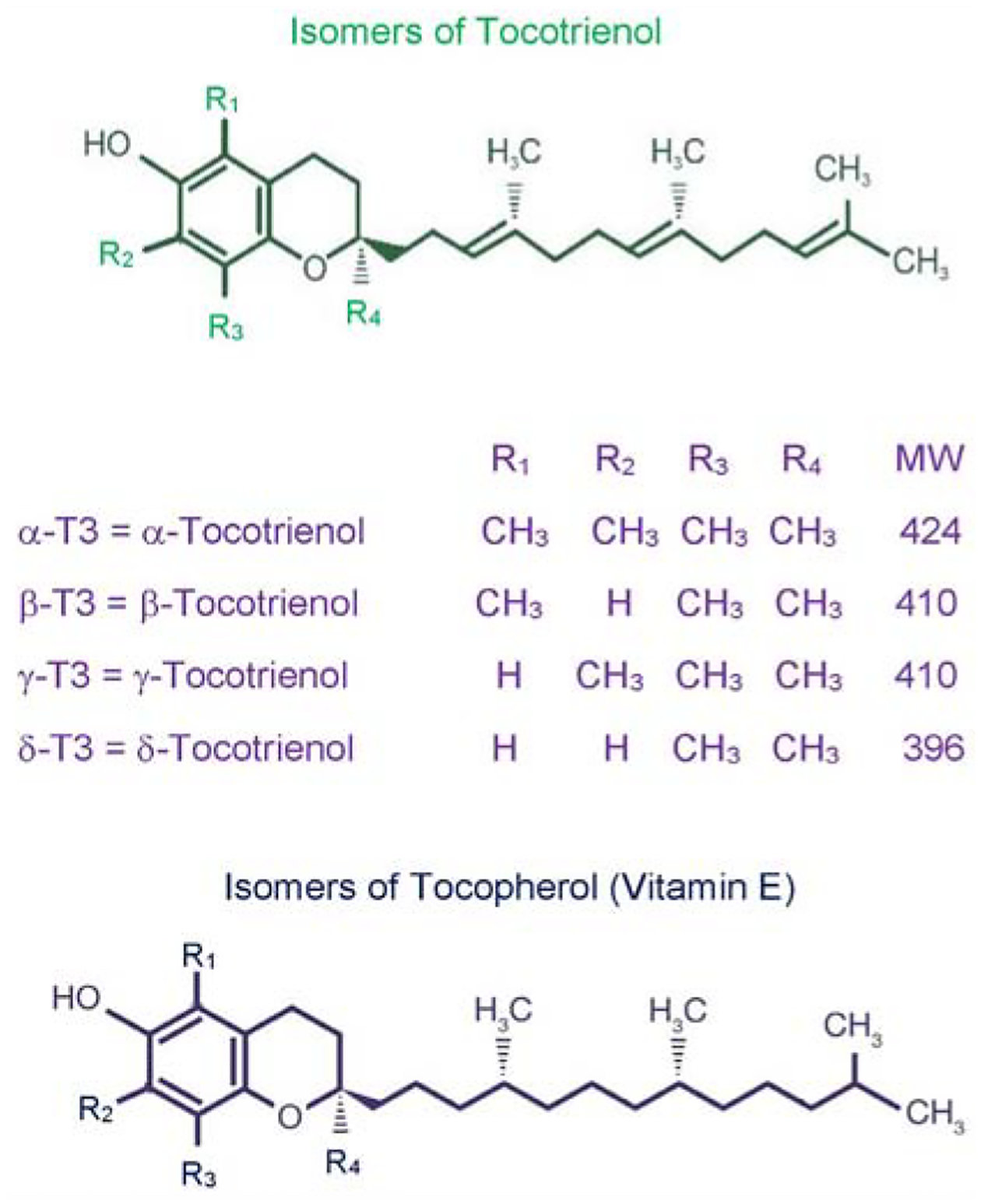
Chemical structures of various isomers of tocotrienol and tocopherol.

Later, we reported the isolation of four isomers (α-, β-, γ-, δ-)-tocotrienols and four isomers of tocopherols (α-, β-, γ-, δ-) from rice bran with hypercholesterolemic, antioxidant, and antitumor properties as shown in (Figure 1) [8]. The highest biological and pharmacological activity was found with δ-tocotrienol followed by γ-tocotrienol, α-tocotrienol, and β-tocotrienol (which does not have any activity) [7]. Tocopherol isomers (vitamin E) do not lower serum cholesterol in animal or human models, rather α-tocopherol attenuates the impact of γ-tocotrienol on hepatic β-hydroxy-β-methylglutaryl coenzyme A reductase activity (the rate-limiting step in de novo cholesterol synthesis)) in chickens as demonstrated by us and others [9,10].

During last three decades, several investigators have published outstanding papers in several areas on the pharmacological properties of various tocotrienols on cardiovascular, cancer, diabetes, metabolic, gastric, skin, osteoporosis, arthritis, and peptic ulcers in humans and several cell-lines as reviewed by Sen et al. [11]. Recently, two other comprehensive reviews on the several biological activities of tocotrienols as hypocholesterolemic, anti-inflammatory, anticancer, antioxidant, neuroprotective, skin protection benefits, bone health and longevity have been published by Wong et al. [12], and Kanchi et al. [13]. These articles also cover the beneficial properties of different isomers of tocotrienol treatment along with possible mechanisms, signaling pathways in breast, prostate, pancreas, rectal cancers in cell lines and humans [12,13]. The present review describes our published results *in vitro* and *in vivo* studies on the impact of tocotrienols and other natural products on inflammation, cardiovascular, cancer, hepatitis C diseases, type 2 diabetes and pharmacokinetics using several cell lines, experimental animal models and human subjects from 2011 to present day. Most of the information described here is based on our published papers during last decade (2011–2021).

All human studies were double-blind, randomized, placebo-controlled trial (RCT). A non-probability convenience sampling technique was used. The protocol of each human study was registered with WHO regional office in Asia (World Health Organization Sri Lanka Clinical Trial Registry, Sri Lanka Center; srilanactr@gmail.com, after ethical approval by the Institutional Review Board of Armed Forces Institute of Pathology (AFIP), Rawalpindi, Pakistan. The registry number and date has been reported in each human study paper. The studies were carried out according to the guidelines provided by the United States Food and Drug Administration (FDA, 2003) at (AFIP), Rawalpindi, and National University of Medical Sciences, Rawalpindi, Pakistan. All participants of human studies have signed an informed consent form before start of the study. All papers were published in refereed journals.

## Anti-Inflammatory and Hypocholesterolemic Properties of Tocotrienols and Other Compounds in Various Experimental Models

**Figures 2:**
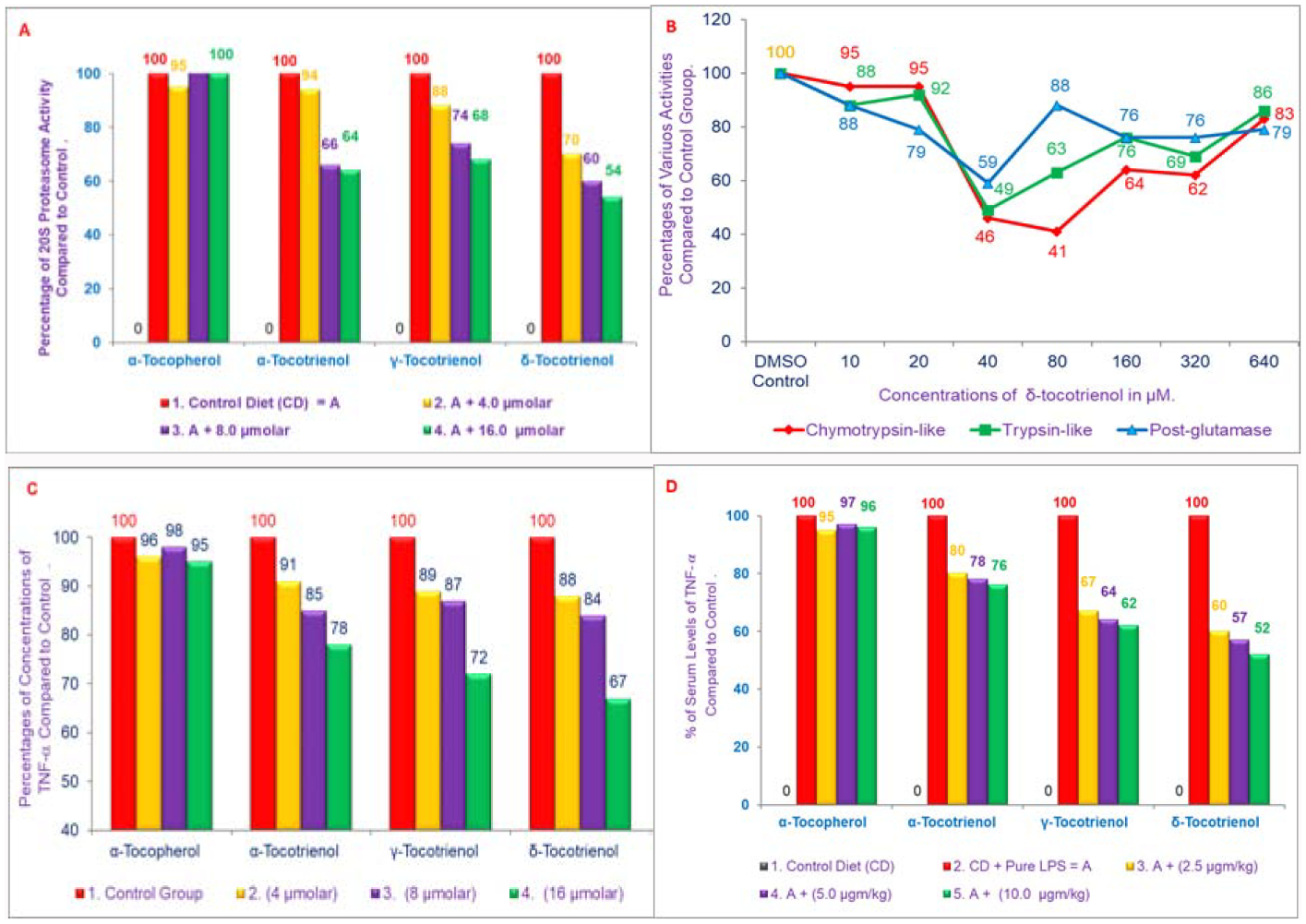
A, B) Effects of various tocols treatments on the chymotrypsin activity of 20S rabbit muscle proteasome (10 μM to 640 μM) of RAW 264.7 cells. A) Chymotrypsin activity was carried out by using synthetic dipeptide substrate III (Suc-Leu-Leu-Val-Tyr-AMC) in buffer pH 7.5, 0.02 M Rabbit muscle proteasome was used. The experimental details were as described in [18]. The mixtures in the plates were assayed according to Promega Protocol. Plates were read with a “Promega Luminator” (fluorescence absorption at excitation =360 nm, and emission =460 nm) to give Relative Luminescence Units (RLV) values [18]. C, D) Effects of various tocols on the synthesis of TNF-α in LPS-stimulated RAW 264.7 Cells and 6-week-old female BALB/c mice. C) The RAW 264.7 cells (500 μL) were adhered for 2 h in all the wells at room temperature. After 2 h, the cells were treated with various concentrations of α-tocopherol, or α-, γ-, or δ-tocotrienols (100 μL) for 1 h, and then the wells were treated with LPS (1 ng/well, 400 μL) for 4 h [18]. D) The BALB/c female mice were acclimatized for 2 wk. Three mice for each concentration were injected Intra-Peritoneal (I.P.) with compounds suspended in 5% triethylamine solution (0.2 L/mouse) one h before LPS challenge. Mice were bled 2 h later to collect serum. The TNF-α assay was carried out by using ELISA kit [18].

**Figures 3:**
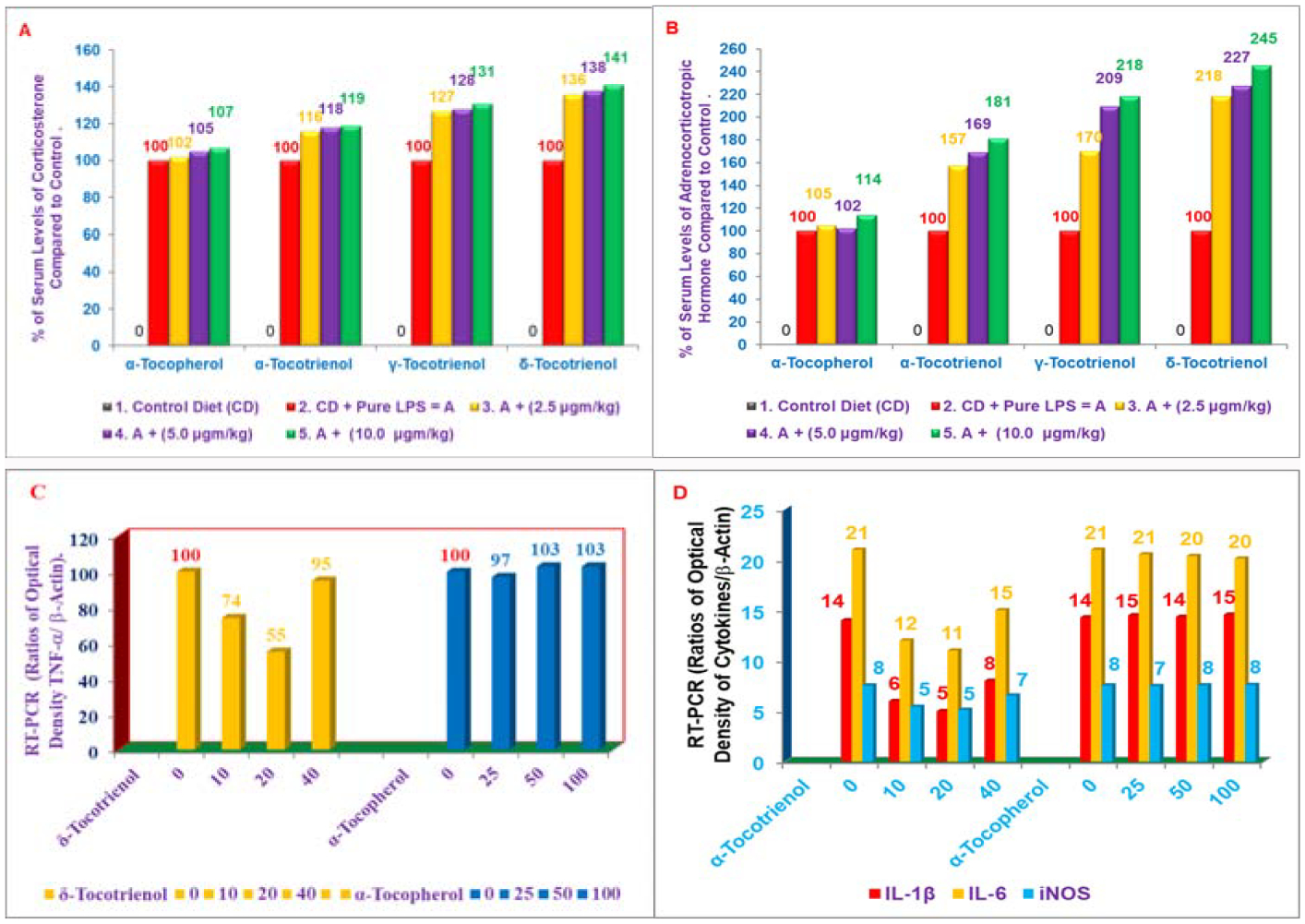
A, B) Effects of tocols on the synthesis of corticosterone and adrenocorticotropic hormone of serum stimulated by LPS in 6-week-old BALB/c female mice: Three mice for each concentration were injected I.P. with tocols in 5% triethylamine solution (0.2 mL/mouse) one h prior to LPS challenge, after conditioning them for 14 days. Mice were bled after 2 h later to collect serum. The estimation of serum levels of corticosterone and adrenocorticotropic hormone were carried according to published procedure (Zuckerman et al. Infectious Immunity [18]. C, D) Effects of α-tocopherol *vs*. δ-tocotrienol on the gene expression of TNF-α, IL-1β, IL-6 and iNOS in LPS-stimulated peritoneal macrophages of 6-week-old BALB/c female mice. The RT-PCR procedure was described in the experimental section. The ratio of relative optical density (cytokines/β-actin) of each treatment for each marker was used to draw these figures [18].

As mentioned earlier, inflammation has been implicated in cardiovascular disease, and the important role of proteasome in the development of inflammation and other macrophage functions has been demonstrated by us [14–16]. Lipopolysaccharide (LPS) was also used as a prototype for inflammation [17]. Tocotrienols are potent hypocholesterolemic agents that inhibit β-hydroxy-β-methylglutaryl coenzyme A reductase activity, which is degraded via the ubiquitin-proteasome pathway. Our results have demonstrated the impact of various tocotrienols (α-, γ-, or δ-tocotrienol; Figure 1) treatments inhibit the chymotrypsin-like activity of 20S rabbit muscle proteasome (>50%) in murine RAW 264.7 cells and BALB/c mice (Figure 2A). Furthermore, chymotrypsin-like, trypsin-like, post-glutamase activities were decreased >40% with low concentrations (<80 μM) and increased gradually (80 μM to 640 μM) in RAW 264.7 cells and BALB/c mice (Figure 2B) [18]. Moreover, tocotrienols showed 9% to 33% inhibition in TNF-α secretion in LPS-stimulated RAW 264.7 cells (Figure 2C). Serum levels of TNF-α with LPS-induced secretion were reduced (20% to 48%) by tocotrienols with the doses of 1 and 10 μg/kg in mice (Figure 2D), and a corresponding rise was observed in serum levels of corticosterone (19% to 41%) and adrenocorticotropic hormone (81% to 145%) (Figure 3A, 3B). The maximum inhibition was observed with δ-tocotrienol (10.0 μg/kg) [18]. The low concentrations of δ-tocotrienols (<20 μM) blocked LPS-induced gene expression of TNF-α, IL-1β, IL-6 and *iNOS* (>40%), and increases with higher concentrations (40 μM) in peritoneal macrophages prepared from BALB/c mice compared to control group (Figures 3C, 3D) [18]. These results represent a novel approach of proteasome modulators, which may lead to the development of new dietary supplements for cardiovascular and other human diseases based on inflammation [18].

**Figure 4:**
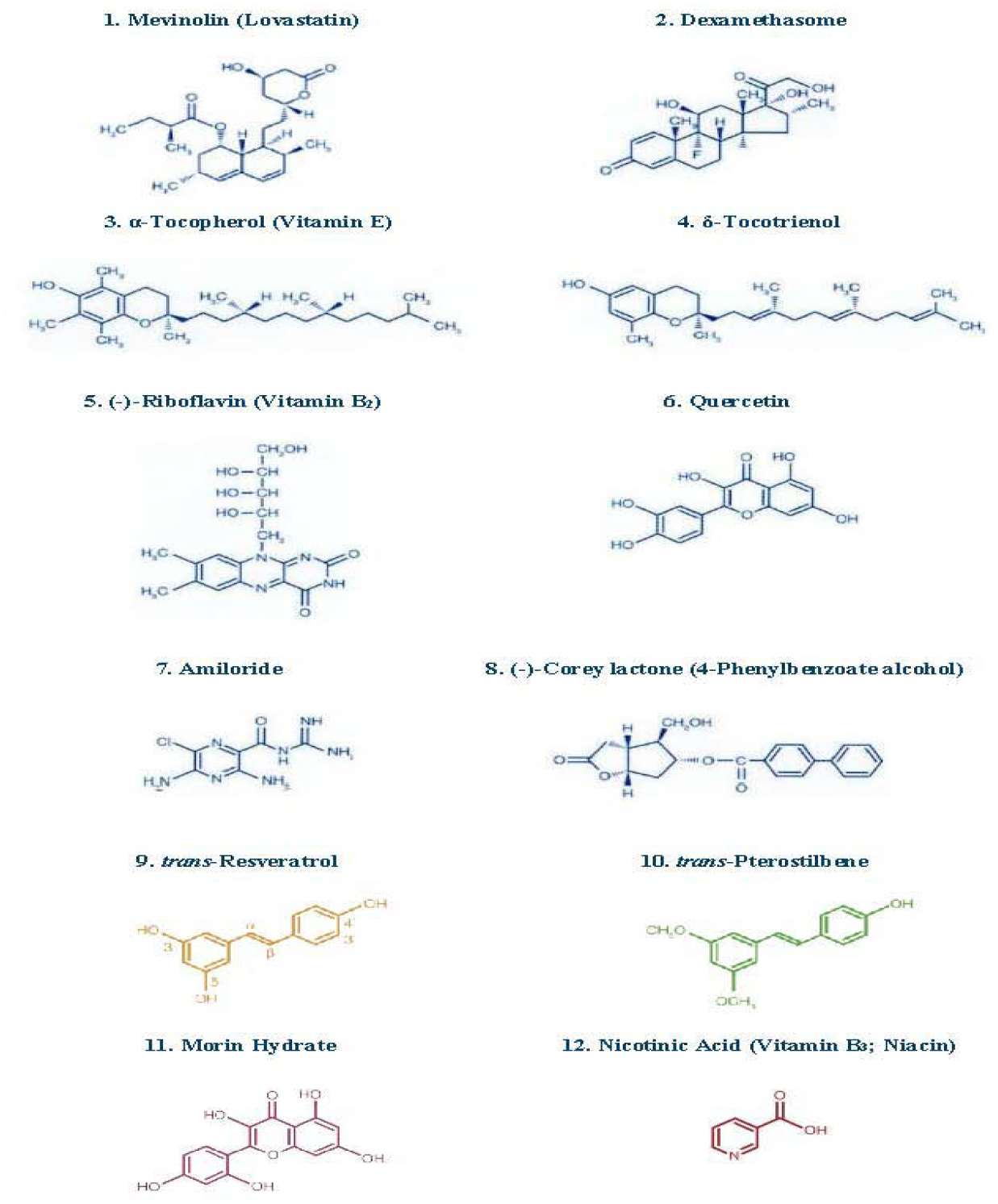
Chemical structures of various compounds used in the studies.

Our results on evaluation of δ-tocotrienol and quercetin on inflammatory markers and lipid parameters was reported in Chickens. It is well known that inflammatory responses to a wide variety of stimuli are largely attributable to up-regulation of the pro-inflammatory transcription nuclear factor kappaB (NF-κB). Specifically, Reactive Oxygen Species (ROS) up-regulate the pro-inflammatory NF-κB transcription factor [19]. The increased transport of NF-κB to the cell nucleus enhances expression of numerous genes encoding proteins that contribute to the inflammatory process, including inducible Nitric Oxide Synthase (*iNOS*), Cyclooxygenase-2 (COX-2), Tumor Necrosis Factors (TNF-α, TNF-β), Interleukins (IL-1, IL-6), chemokines (IL-8, MCP1, and MIP1α), Activator Protein-1 (AP-1) and adhesion factors (ICAM, and VCAM). Several of the proteins encoded by genes that are up-regulated by NF-κB are also potent NF-κB activators [19]. A series of in vitro tests confirmed the strong anti-inflammatory activities of known inhibitors of NF-κB activation (δ-tocotrienol, quercetin, riboflavin, (−) Corey lactone, amiloride, and dexamethasone; Figure 4 [20]). As it was demonstrated that δ-Tocotrienol suppresses β-Hydroxy-β-Methylglutaryl Coenzyme A (HMG-CoA) reductase activity, and concomitantly lowers serum total and LDL cholesterol levels [8]. The results of above compounds (Figure 4) were reported in an avian model anticipating that a dietary additive combining δ-tocotrienol with quercetin, riboflavin, (−) Corey lactone, amiloride, or dexamethasone would yield greater reductions in serum levels of total cholesterol, LDL-cholesterol, and inflammatory markers (Tumor Necrosis Factor-α [TNF-α], and Nitric Oxide [NO]), than that attained with the individual compounds [21].

**Figures 5:**
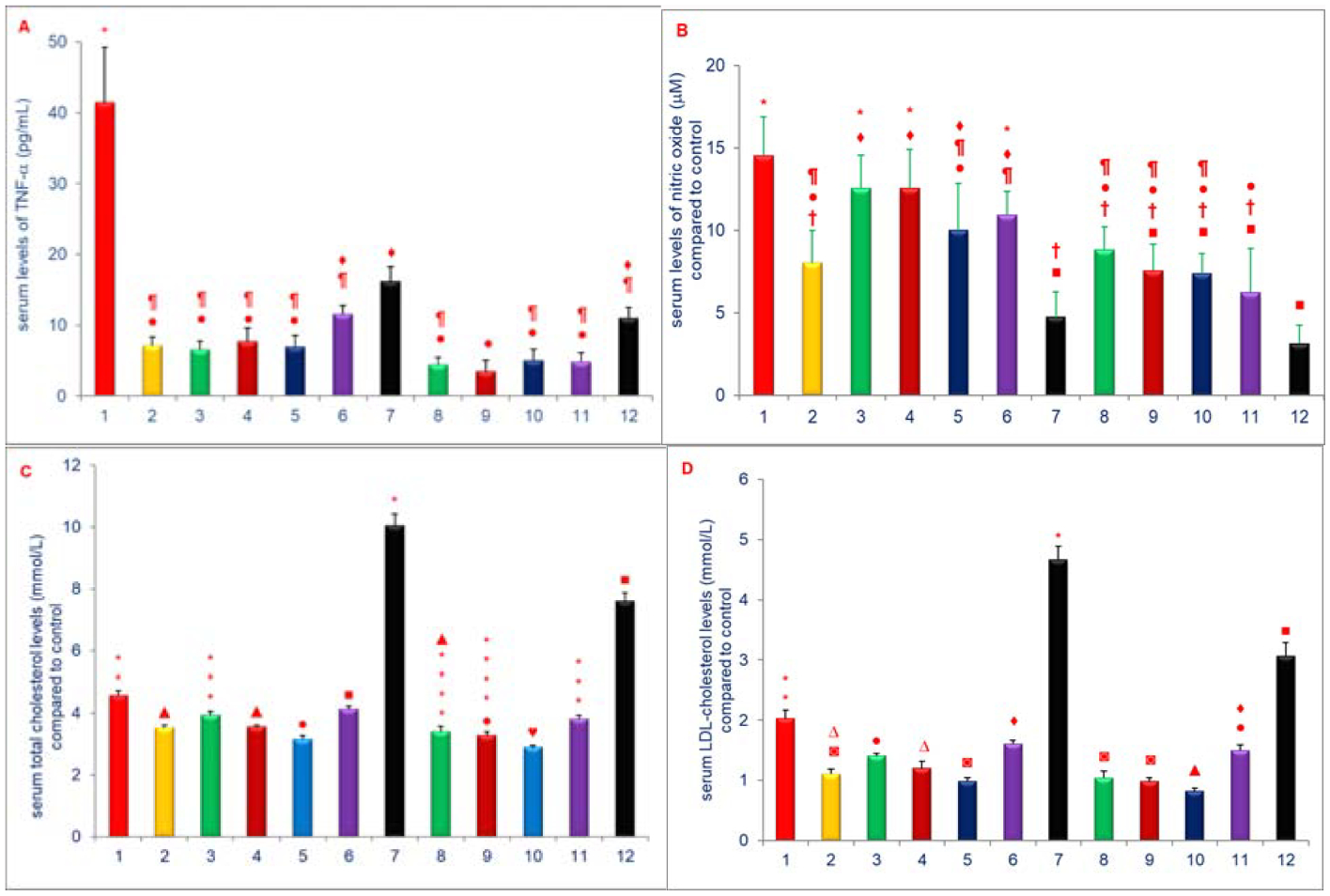
A-D) Effects of dietary supplements on serum levels of TNF-α, NO, total & LDL-cholesterol of 5-week-old female chickens: The 5-weeks old female chickens were fed diets supplemented diets with 1. Control diet; 2. δ-tocotrienol (δ-T3); 3. Quercetin; 4. Riboflavin; 5. (−) Corey lactone; 6. Amiloride; 7. Dexamethasone; 8. δ-T3+ quercetin; 9. δ-T3+ riboflavin; 10. δ-T3+(−) Corey lactone; 11. δ-T3+ amiloride; 12. δ-T3+ dexamethasone for 4-weeks. The estimations of TNF-α (5A), NO (5B), total cholesterol (5C) and LDL-cholesterol (5D) were estimated as described in Experimental section [21]. Data expressed as means ± SD, n=6 chickens per group. The value for the control group was the average of 3 control groups. Values in columns not sharing a common symbol were significantly different at P<0.05 [21].

In these series of experiments, the control diet was supplemented individually with compounds mentioned above (Figure 4) and fed to chickens for 4-weeks, the riboflavin, (−) Corey lactone, δ-tocotrienol, and quercetin produced small reductions in body weight gains as compared to control. Whereas dexamethasone significantly and markedly reduced weight gain (>75%) compared to control [21]. The serum levels of TNF-α and NO were decreased 61% to 84% and 14% to 67%, respectively in chickens fed diets supplemented with δ-tocotrienol, quercetin, riboflavin, (−) Corey lactone, amiloride, or dexamethasone as compared to controls (Figure 5A, 5B). Furthermore, significant decrease in the levels of serum total and LDL-cholesterol was observed with δ-tocotrienol, quercetin, riboflavin and (−) Corey lactone (13% to 57%; Figure 5C, 5D), and these levels were 2-fold higher in dexamethasone treated chickens as compared to controls [21]. Moreover, the combination of δ-tocotrienol with the other compounds yielded even lower levels of these markers as compared to individual components [21]. Exceptions were significantly lower total and LDL cholesterol and triglycerides values attained with the δ-tocotrienol + (−) Corey lactone treatment and the significantly lower triglycerides value attained with the δ-tocotrienol + riboflavin treatment. Thus δ-tocotrienol attenuated the lipid-elevating impact of dexamethasone and potentiated the triglycerides lowering impact of riboflavin (Figure 5C, 5D) [21].

Microarray analyses of liver samples identified 62 genes whose expression was either up-regulated (39 genes) or down-regulated (23 genes) by all compounds suggesting common impact on serum TNF-α and NO levels (Table 1). The microarray analyses genes involved with inflammation further identified up-regulated genes (ubiquitin protein lipase E3C, suppressor of cytokine signaling 1, nuclear factor kappaB, interferon γ receptor, type 1) and down-regulated (proteasome activator subunit 4, tumor necrosis factor superfamily, protein kinase), whose expression was differentially impacted by these compounds in heat map [21], and shown to lower serum lipid levels and dexamethasone, associated with markedly elevated serum lipids (Table 1) [21]. The rest of the genes involved with ageing, cardiovascular disease and cancer are outlined in Table 1 [21]. These results described for the first time that anti-inflammatory effects of δ-tocotrienol, quercetin, riboflavin, (−) Corey lactone, amiloride, and dexamethasone on serum TNF-α and NO levels. Serum TNF-α levels decreased by >60% by each of the experimental compounds. Additionally, all the treatments except for dexamethasone resulted in lower serum total cholesterol, LDL-cholesterol, and triglycerides levels. The impact of above-mentioned compounds on the factors evaluated herein was increased when combined with δ-tocotrienol, thus δ-tocotrienol is the most potent inhibitor for inflammation [21].

**Figures 6:**
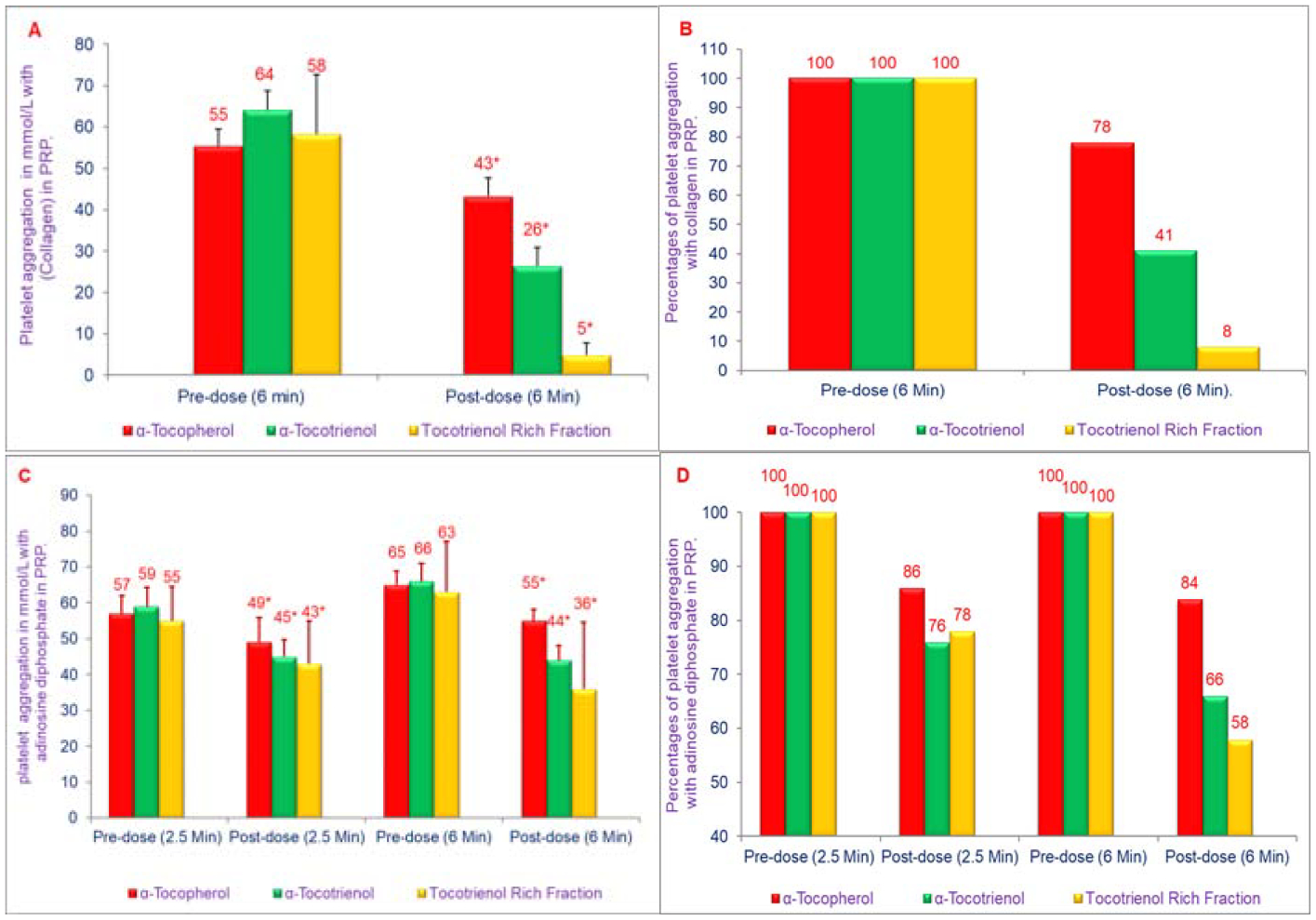
A, B, C, D) Effects of α-tocopherol, α-tocotrienol, and Tocotrienol Rich Fraction (TRF) on collagen-induced or adenosine diphosphate-induced platelet aggregation: α-Tocopherol (250 μg), α-tocotrienol (250 μg) or TRF (250 μg) were dissolved in polyethylene glycol (1 mL) solvent and plasma (40 mL) was warmed to 50°C (20 min) prior to injection, and administered over 30 seconds through a femoral arterial line to anesthetized dogs. Collagen (12.5 μmol/mL) or adenosine diphosphate (20 μmol/mL) in a volume of 32 μL was added to 400 μL of PRP that had been incubated at 37°C for 2 min. The extent of collagen- or adenosine diphosphate-induced platelet aggregation was quantitated by measuring the percent maximal aggregation after 6 min Data are expressed as means ± SD, n=3 per treatment. 6A, 6C is based on raw values and 6B, 6D is based on percentages compared to their respective pre-dose and post-dose control values. An asterisk indicates significant differences at P<0.001 or P<0.05 for each treatment compared to respective controls [22].

Tocotrienols have potent anti-inflammatory, antioxidant, anticancer, hypocholesterolemic, and neuroprotective properties as described earlier ([8,11–13]; Figure 1). The results of our study have described the extent to which tocotrienols inhibit platelet aggregation and reduce coronary thrombosis, a major risk factor for stroke in humans. The comparative effects of α-tocopherol, α-tocotrienol, or Tocotrienol Rich Fraction (TRF; a mixture of α- + γ- + δ-tocotrienols) on *in vivo* platelet thrombosis and *ex vivo* Platelet Aggregation (PA) has been described after intravenous injection in anesthetized dogs, using a mechanically stenosed circumflex coronary artery model (Folts’ cyclic flow model) [22]. The collagen induced Platelet Aggregation (PA) in platelet rich plasma decreased markedly after treatment with α-tocotrienol (59%) and TRF (92%). α-Tocopherol treatment was less effective, producing only a 22% decrease in PA. Adenosine Diphosphate-induced (ADP) PA was also decreased after treatment with α-tocotrienol (34%) and TRF (42%) as shown in Figures 6A–6D) [22].

The results also indicated that intravenously administered tocotrienols were significantly better than tocopherols in inhibiting Cyclic Flow Reduction (CFRs), a measure of acute platelet-mediated thrombus formation. Tocotrienols given intravenously (10 mg/kg), abolished CFRs after a mean of 68 min (range 22 min to 130 min), and this abolition of CFRs was sustained throughout the monitoring period (50 min to 160 min) [21]. The pharmacokinetic results indicated that treatment with α-tocopherol increased levels of total tocopherols in post-treatment *vs*. pre-treatment specimens (57 μg/mL *vs*. 18 μg/mL in plasma and 42 μg/mL *vs*. 10 μg/mL in platelets (Table 2A). However, treatment with α-tocopherol resulted in slightly decreased levels of tocotrienols in post-treatment *vs*. pre-treatment samples (1.4 μg/mL *vs*. 2.9 μg/mL in plasma and 2.3 μg/mL *vs*. 2.8 μg/mL in platelets (Table 2A) [22].

However, treatment with α-tocotrienol increased levels of both tocopherols and tocotrienols in post-treatment *vs*. pre-treatment samples (tocopherols, 45 μg/mL *vs*. 10 μg/mL in plasma and 28 μg/mL *vs*. 5 μg/mL in platelets; tocotrienols, 2.8 μg/mL *vs*. 0.9 μg/mL in plasma and 1.28 μg/mL *vs*. 1.02 μg/mL in platelets (Table 2B). The TRF treatment also increased levels of tocopherols and tocotrienols in post-treatment *vs*. pre-treatment samples (tocopherols, 68 μg/mL *vs*. 20 μg/mL in plasma and 31.4 μg/mL *vs*. 7.9 μg/mL in platelets, tocotrienols, 8.6 μg/mL *vs*. 1.7 μg/mL in plasma and 5.8 μg/mL *vs*. 3.9 μg/mL in platelets, (Table 2C) [22]. These results indicated that intravenously administered tocotrienols inhibited acute platelet-mediated thrombus formation, and collagen and ADP-induced platelet aggregation. Tocotrienol treatment induced increase in α-tocopherol levels of 2-fold and 4-fold in plasma and platelets, respectively. Interestingly, tocotrienol treatment induced a less pronounced increase in the levels of tocotrienols in plasma and platelets, suggesting that intravenously administered tocotrienols may be converted to tocopherols. Tocotrienols, given intravenously, could potentially prevent pathological platelet thrombus formation and provide a therapeutic benefit in conditions such as stroke and myocardial infarction [22].

It is well known that as humans age, they are at increased risk for a variety of age-associated diseases such as cardiovascular, arthritis, diabetes, obesity, dementia, cancer, and atherosclerosis [23]. Over the past decade, it has become increasing evident that dysregulated immune function, leading to chronic inflammation, which contributes to the pathogenesis of several of these age-associated disease [24].

**Figures 7:**
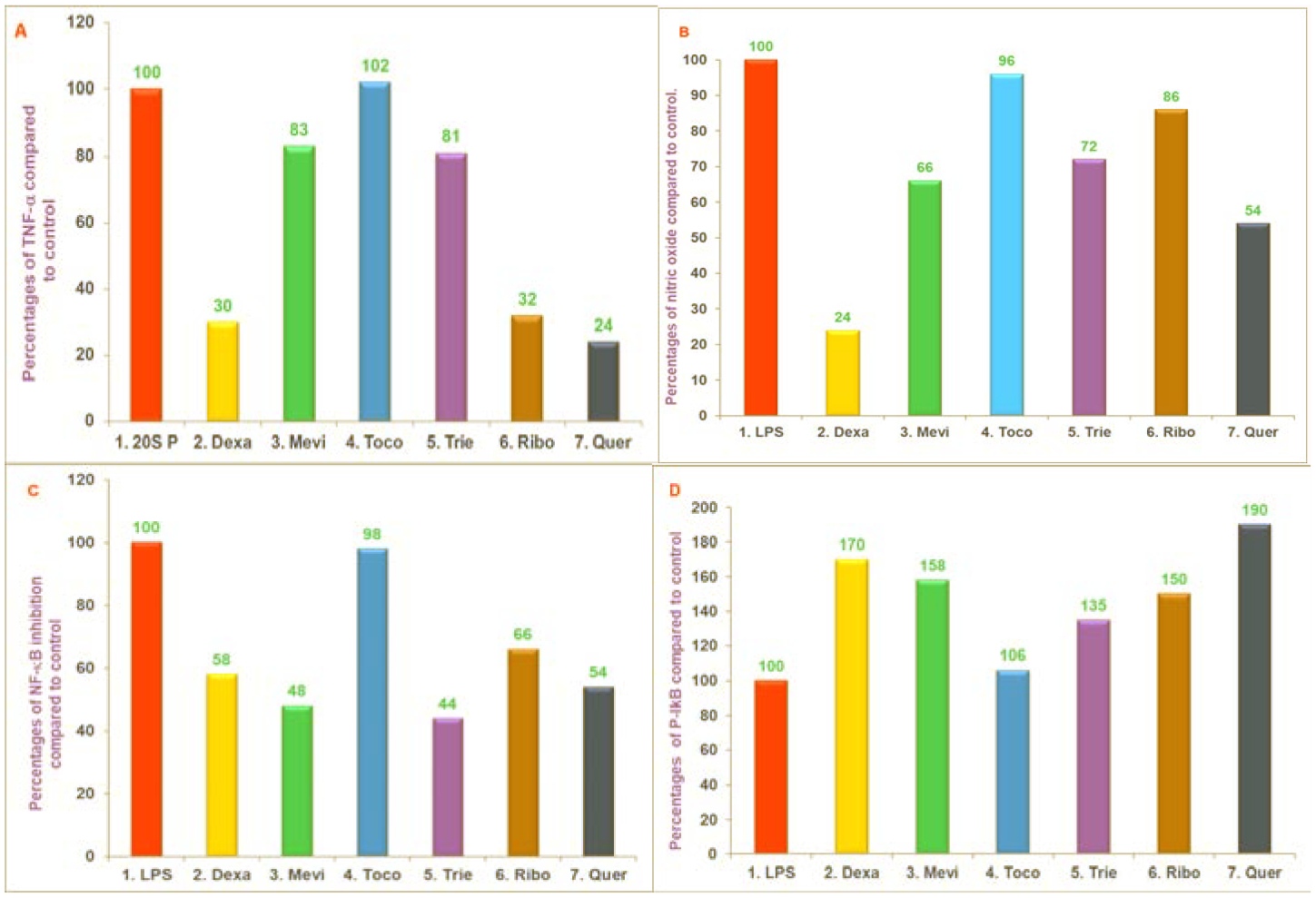
A, B, C, D) The effect of proteasome inhibitors on TNF-α secretion, NO production, translocation of NF-κB to the nucleus and intracellular levels of P-IκB protein in LPS-stimulated RAW 264.7 cells. The RAW 264.7 cells were treated for 1 hour with tris +20 SP in 0.5% DMSO (control), dexamethasone (10 μM), mevinolin (20 μM), α-tocopherol (100 μM), 5. δ-tocotrienol (10 μM), riboflavin (40 μM), or quercetin-HCL (40 μM). All the wells were challenged with LPS (10 ng/well; 400 μL) and incubated at 37°C 5% CO2 for 4 h. Supernatants were assayed for TNF-α secretion by ELISA kit (7A), or for production of NO by measuring the amount of nitrite using the Griess reagent (7B), or NF-κB activation was measured using “Electronic Mobility Shift Assay” (EMSA) (7C) and P-IκB-α levels were measured by Western Blot analysis (7D). The treatments 1–7 correspond to: 1. Control (tris+20 SP in 0.5% DMSO); 2. Dexamethasone (10 μM); 3. Mevinolin (20 μM); 4. α-tocopherol (100 μM); 5. δ-tocotrienol (10 μM); 6. Riboflavin (40 μM); 7. Quercetin-HCL (40 μM). Experiments detail [25].

We have recently reported that the proteasome is a pivotal regulator of inflammation, which modulates the induction of inflammatory mediators such as TNF-α, IL-1, IL-6, and Nitric Oxide (NO) in response to a variety of stimuli [24]. The impact of various proteosome inhibitors (Figure 4) resulted in suppression of TNF-α, NO and gene suppression of TNF-α and *iNOS* of mRNA, by LPS-stimulated macrophages obtained from several sources. Further, the mechanisms by which these agents suppress secretion of TNF-α and NO production have also been reported [25]. The effects of mevinolin, dexamethasone, α-tocopherol, δ-tocotrienol, riboflavin, quercetin amiloride and (−) Corey lactone have been reported in RAW 264.7 cells, and peritoneal macrophages obtained from C57BL/6, BALB/c, proteasome double subunits knockout LMP7MECL-1^−/−^ and peroxisome proliferator-activated receptor-α^−/−^ (PPAR-α^−/−^ knockout mice. There was significant reduction in chymotrypsin-like activity of the 20S rabbit muscle proteasomes with dexamethasone (31%), mevinolin (19%), δ-tocotrienol (28%), riboflavin (34%), and quercetin (45%). Moreover, quercetin, riboflavin, and δ-tocotrienol inhibited chymotrypsin-like, trypsin-like, and post-glutamase activities in RAW 264.7 cells [25]. These compounds also inhibited LPS-stimulated NO production and TNF-α secretion, blocked the degradation of P-IκB protein, and decreased activation of NF-κB in RAW 264.7 cells (Figures 7A–7D) [25]. All these compounds significantly inhibited NO production (30% to 60%) by LPS-induced thioglycolate-elicited peritoneal macrophages derived from all four strains of mice.

**Figures 8:**
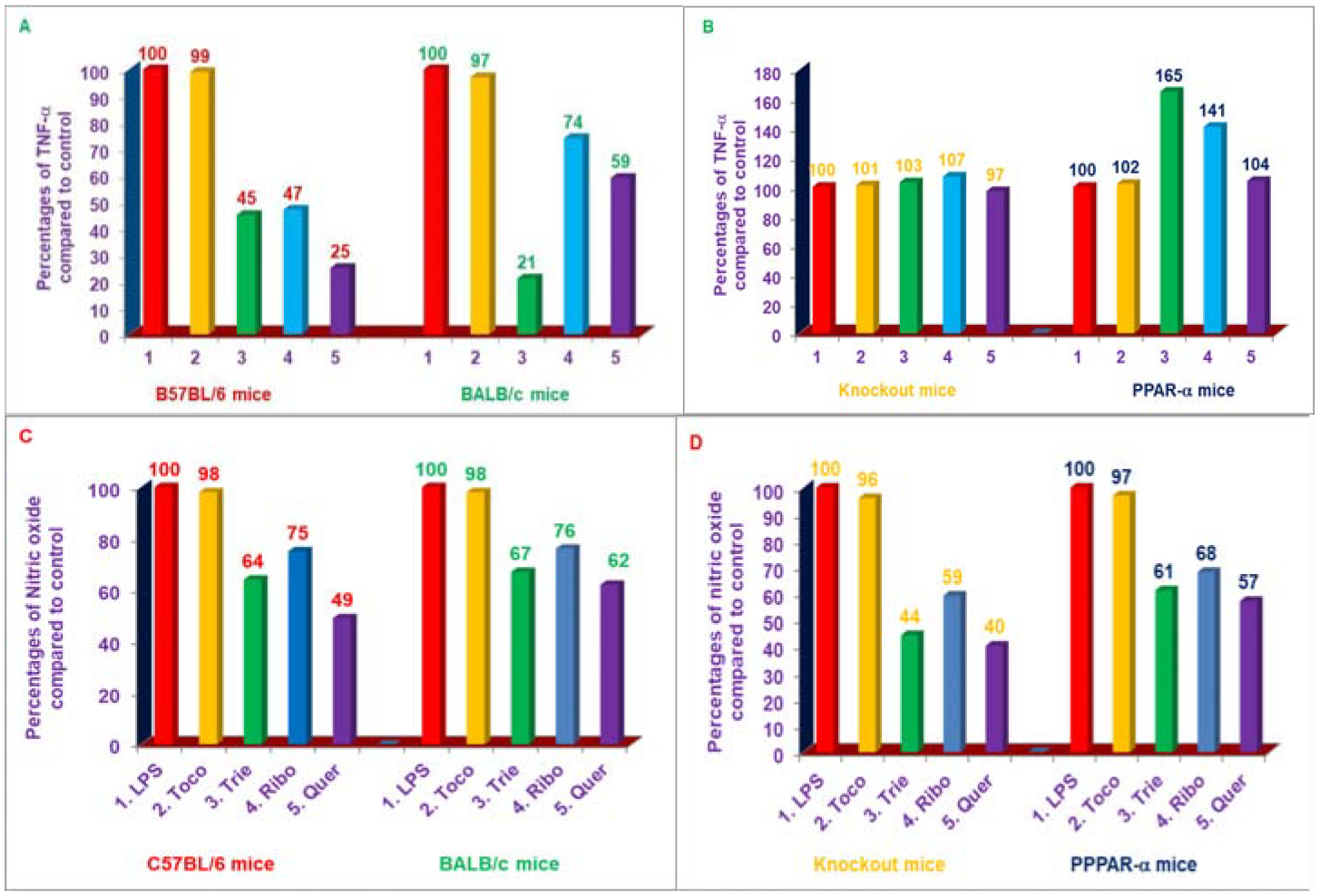
A, B, C, D) Effects of various compounds on the secretion of TNF-α in LPS-stimulated peritoneal macrophages of C57BL/6 versus BALB/c and double subunits knockout (LMP7/MECL-1-) *vs*. PPAR-α^−/−^ 8-week-old female mice. The effectiveness of these compounds as potent inhibitors for the inhibition of secretion of TNF-α or NO production of dexamethasone, mevinolin, α-tocopherol, δ-tocotrienol, riboflavin, or quercetin-HCL were tested in 6-week-old female C57BL/6 and BALB/c mice (5 of each), which were acclimatized for two weeks to new environment, then thioglycolate-elicited peritoneal macrophages were prepared from these 8-week-old mice as described previously [25]. The macrophages (1 × 10^7^/well) were treated with 100 μL containing dexamethasone, 10 μM; mevinolin, 20 μM; δ-tocotrienol, 10 μM; α-tocopherol, 100 μM; riboflavin, 40 μM; or quercetin-HCL, 40 μM for I h, followed by stimulation with LPS (10 ng/mL) of each treatment. The detail of assay mixtures was described in Experimental section [25]. Data are means ± SD, n=5 per treatment, and triplicate analyses of each sample. The treatments 1–7 correspond to: 1. Control (macrophages+LPS+0.2% DMSO); 2. α-tocopherol; 3. δ-tocotrienol; 4. Riboflavin; 5. Quercetin-HCL [25].

These five compounds also suppressed LPS-induced secretion of TNF-α in macrophages obtained from C57BL/6 and BALB/c mice [25]. The TNF-α secretion, however, was not suppressed by any of these three proteasome inhibitors tested (δ-tocotrienol, riboflavin, and quercetin) with LPS-stimulated macrophages from LMP7/MECL-1^−/−^ and PPAR-α^−/−^ knockout mice. Results of gene expression for TNF-α and *iNOS* were generally consistent with results obtained for TNF-α protein and NO production as observed with four strains of mice (Figures 8A–8D) [25].

These results have indicated that δ-tocotrienol, riboflavin, and quercetin inhibit NO production in LPS-stimulated macrophages of all four strains of mice, and TNF-α secretion only in LPS-stimulated macrophages of C57BL/6 and BALB/c mice [25]. The mechanism for this inhibition appears to be decreased proteolytic degradation of P-IκB protein due to the inhibition of proteasome, resulting in decreased translocation of activated NF-κB to the nucleus, and depressed transcription of gene expression of TNF-α, and *iNOS*. Furthermore, these naturally occurring proteasome inhibitors appear to be relatively potent inhibitors of multiple proteasome subunits in inflammatory proteasomes. Consequently, these agents could potentially suppress the production of inflammatory mediators in ageing humans, thereby decreasing the risk of developing a variety of age-related diseases [25].

In recent years, the concept that age-associated diseases such as cardiovascular, cancer, dementia might be attributable, in part, to dysregulated inflammatory responses has been the subjects of extensive discussion [26]. Changes in immune function believed to contribute to a variety of age-related diseases, which have been associated with increased production of Nitric Oxide (NO). It was recently reported that dexamethasone, mevinolin, quercetin, δ-tocotrienol, and riboflavin (Figure 4); can inhibit Lipopolysaccharide (LPS)-induced NO production *in vitro* in RAW 264.7 cells and in thioglycolate-elicited peritoneal macrophages derived from four strains of mice (C57BL/6, BALB/c, LMP7/MECL-1^−/−^ and PPAR-α^−/−^ knockout mice) [25]. The potential results of the effects of diet supplementation with naturally occurring compounds (δ-tocotrienol and quercetin) on LPS-stimulated production of NO, TNF-α, and other pro-inflammatory cytokines involved in the ageing process have been reported. Young (4-week-old) and senescent mice (42-week-old) were fed control diet with or without quercetin (100 ppm), δ-tocotrienol (100 ppm), or dexamethasone (10 ppm; included as positive control for suppression of inflammation) for 4-weeks (Figure 4). The thioglycolate-elicited peritoneal macrophages were collected, stimulated with LPS, LPS plus Interferon-β (IFN-β), or LPS plus Interferon-γ (IFN-γ), and inflammatory responses assessed as measured by production of NO and TNF-α, mRNA down-regulation for TNF-α, and *iNOS* genes, and microarray analysis [27].

**Figures 9:**
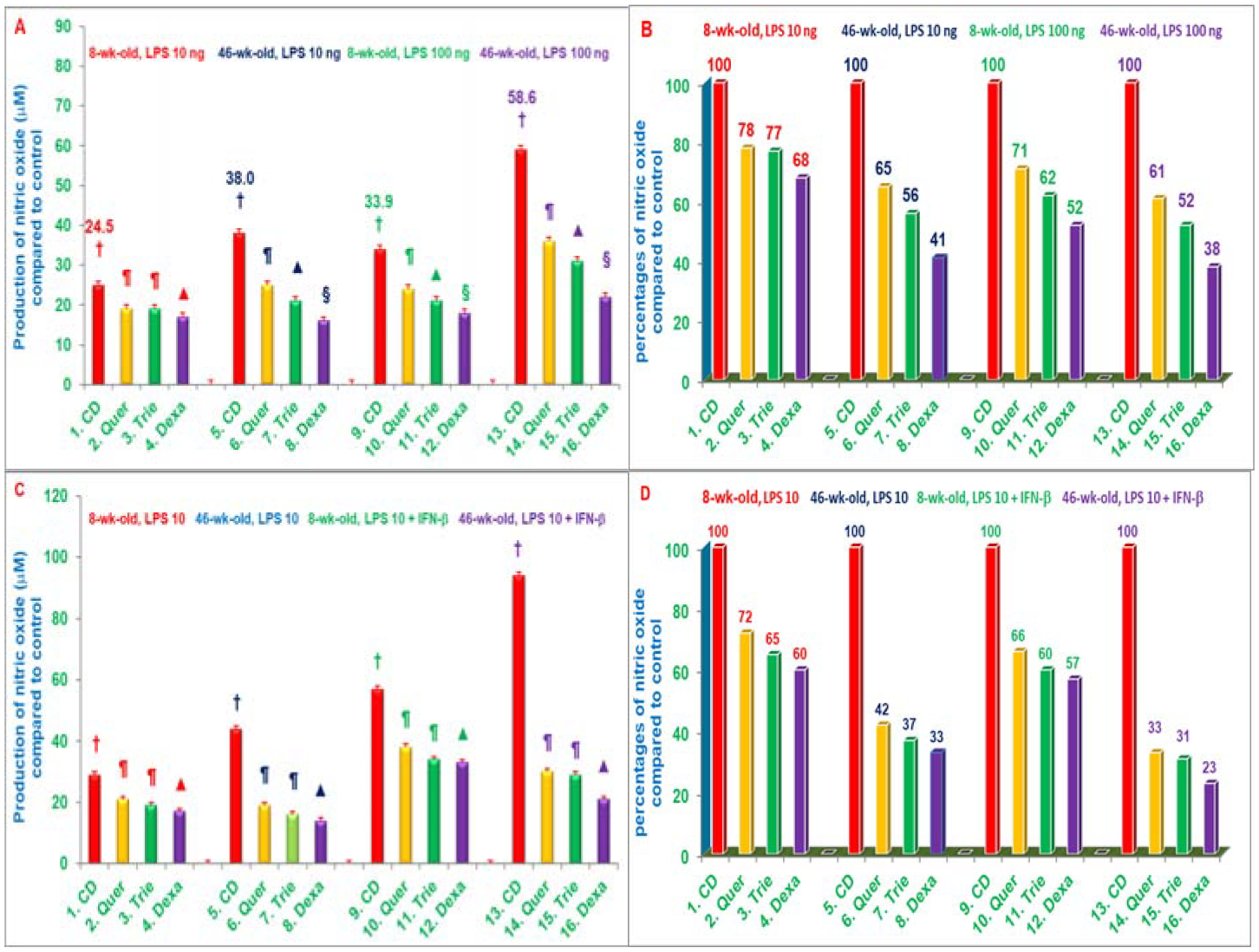
A, B, C, D) Effects of dietary supplementation with various compounds on the production of nitric oxide (μM) by LPS-stimulated thioglycolate-elicited peritoneal macrophages with or without IFN-β. 8-weeks-old and 42-weeks-old C57BL/6 male mice were fed control diet or control diets supplemented with quercetin, δ-tocotrienol or dexamethasone with or without IFN-β for 4 weeks. After this, thioglycolate-elicited peritoneal macrophages were collected, adhered to the bottom of 12 well plates (1 × 10^7^ cells/well in 1.0 mL medium) for 4 h, and then washed with medium three times. Cells were then treated with LPS (10 ng/well or 100 ng/well) for 18 h at room temperature, the mixtures were centrifuged at 2000 rpm for 20 min, and supernatants were collected and estimated production of NO using Griess reagent. Data represent means ± SD; n=3 mice per group with triplicate analysis of each sample. Values in columns with different symbols differ at P< 0.05. Treatments with IFN-β (9C, D) or without IFN-β (9 A, B) 1–16 correspond to: 1–4=8-weeks-old or 5–8=46-weeks-old mice treated with 10 ng/well LPS; 9–12=8-weeks-old or 13–16=46-weeks-old mice treated with 100 ng/well LPS. CD: Control Diet; Quer: Quercetin; Trie: δ-Tocotrienol; Dexa: Dexamethasone [27]

The results of thioglycolate-elicited peritoneal macrophages prepared after four weeks of feeding of these mice, and then challenged with LPS (10 ng or 100 ng) resulted in increases of 55% and 73%, respectively in the production of NO of 46-week-old compared to 8-week-old mice fed control diet alone (respective control groups), without affecting the secretion of TNF-α among these two groups [27]. However, macrophages obtained after feeding with quercetin, δ-tocotrienol, and dexamethasone significantly inhibited (30% to 60%) the LPS-stimulated NO production, compared to respective control groups (Figures 9A–9D). There was a 2-fold increase in the production of NO, when LPS-stimulated macrophages of quercetin, δ-tocotrienol, or dexamethasone were also treated with IFN-β or IFN-γ compared to respective control groups. It has been also demonstrated that NO levels and *iNOS* mRNA expression levels were significantly higher in LPS-stimulated macrophages from senescent (0.69 *vs*. 0.41), compared to young mice. In contrast, age did not appear to impact levels of TNF-α protein or mRNA expression levels (0.38 *vs*. 0.35) in LPS-stimulated macrophages (Table 3) [27]. These results have demonstrated that quercetin and δ-tocotrienols inhibit the LPS-induced NO production *in vivo*. The microarray RNAs analyses, followed by pathway analyses indicated that quercetin or δ-tocotrienol inhibit several LPS-induced gene expressions of several ageing and pro-inflammatory genes (IL-1β, IL-1α, IL-6, TNF-α, IL-12, *iNOS*, VCAM1, ICAM1, COX2, IL-1RA, TRAF1 and CD40) as shown in Table 3 [27]. The NF-κB pathway regulates the production of NO and inhibits the pro-inflammatory cytokines involved in normal and ageing process. The *ex vivo* results confirmed the earlier *in vitro* findings. These results of inhibition of NO production by quercetin and δ-tocotrienol may be of clinical significance treating several inflammatory diseases, including ageing process [27].

It was reported earlier that altered immune function during ageing results in increased production of Nitric Oxide (NO) and other inflammatory mediators. Recently, it was also reported that NO production was inhibited by naturally occurring quercetin, δ-tocotrienol, and riboflavin in Lipopolysaccharide (LPS)-stimulated RAW 264.7 cells, and thioglycolate-elicited peritoneal macrophages from C57BL/6 mice (Figure 4). In our continuous effort to find more potent, non-toxic, commercially available, naturally occurring compounds that suppress inflammation, the results of study have reported the inhibition of NF-κB activation and NO, TNF-α, IL-6, IL-1β, and *iNOS* gene expression by trans-resveratrol, transpterostilbene, morin hydrate, and nicotinic acid in LPS-induced RAW 264.7 cells and thioglycolate-elicited peritoneal macrophages from C57BL/6 and BALB/c mice [28].

These results have also demonstrated that resveratrol, pterostilbene, and morin hydrate caused significant inhibition (>70% to 90%) in the activities of chymotrypsin-like, trypsin-like, and post-acidic (post-glutamase) proteasome sites in RAW 264.7 cells at a dose of only 20 μM (Table 4) [27]. These compounds inhibited the production of NO by RAW-264.7 cells stimulated with LPS alone (>40%), or LPS + Interferon-γ (IFN-γ; >60%. Furthermore, resveratrol, pterostilbene, morin hydrate, and quercetin suppressed secretion of TNF-α (>40%) in LPS-stimulated RAW 264.7 cells and suppressed NF-B activation (22% to 45%) in LPS-stimulated HEK293T cells [28]. Moreover, these compounds also significantly suppressed LPS-induced gene expression of TNF-α, IL-1β, IL-6, and *iNOS* genes in RAW 264.7 cells (Table 5), and in thioglycolate-elicited peritoneal macrophages from C57BL/6 and BALB/c mice [28].

These results have demonstrated that resveratrol and pterostilbene are particularly potent compounds that suppress expression of genes, and production of inflammatory products in LPS-stimulated RAW 264.7 cells, and macrophages from C57BL/6 and BALB/c mice [27]. Resveratrol and pterostilbene which are present in grapes, blueberries, and red wine, have been implicated as contributing factors to the lower incidence of cardiovascular disease in the French population, despite their relatively high dietary fat intake. Consequently, it appears likely that the beneficial nutritional effects of resveratrol and pterostilbene are due at least in part, to their ability to inhibit NF-B activation by the proteasome inhibitors, thereby suppressing activation of pro-inflammatory cytokines and *iNOS* genes, resulting in decreased production of TNF-α, IL-1β, IL-6, and NO levels, in response to inflammatory stimuli. This was the first report that has demonstrated that resveratrol and pterostilbene act as proteasome inhibitors, thus providing a mechanism for their anti-inflammatory effects in mouse cells [28].

## Hypocholesterolmic and Anti-Inflammatory Properties of Tocotrienols and Other Compounds in Humans

It was earlier reported, the anti-inflammatory properties of resveratrol, pterostilbene, morin hydrate, quercetin, δ-tocotrienol, riboflavin in a variety of experimental animal models, and these compounds act by inhibiting proteasomal activity [25]. As reported earlier that serum Nitric Oxide (NO) levels increase with age in humans, and we hypothesized that combined cholesterol-lowering and inflammation-reducing properties of resveratrol, pterostilbene, morin hydrate, quercetin, δ-tocotrienol, riboflavin, and nicotinic acid (Figure 4) would reduce cardiovascular risk factors in humans when used as nutritional supplements with, or without, other dietary changes.

The elderly human subjects were divided into two groups based on total serum cholesterol levels. Initial total serum cholesterol levels were normal in group #1 and elevated in group #2 subjects, respectively, and after establishing the Baseline serum NO, C-Reactive Protein (CRP), γ-Glutamyltransferase (γ-GT) activity, uric acid, Total Antioxidant Status (TAS), total cholesterol, HDL-cholesterol, LDL-cholesterol, and triglycerides levels [29]. Group 1 subjects were administered nutritional supplementation with one of two different combinations (NS-7=25 mg of each, resveratrol, pterostilbene, quercetin, δ-tocotrienol, nicotinic acid, morin hydrate or NS-6=morin hydrate replaced with quercetin, 50 mg/capsule). Group 2 subjects also received these nutritional supplements (two capsules/d), including an AHA Step-1 diet for group 2 subjects for 4-weeks [29].

**Figures 10:**
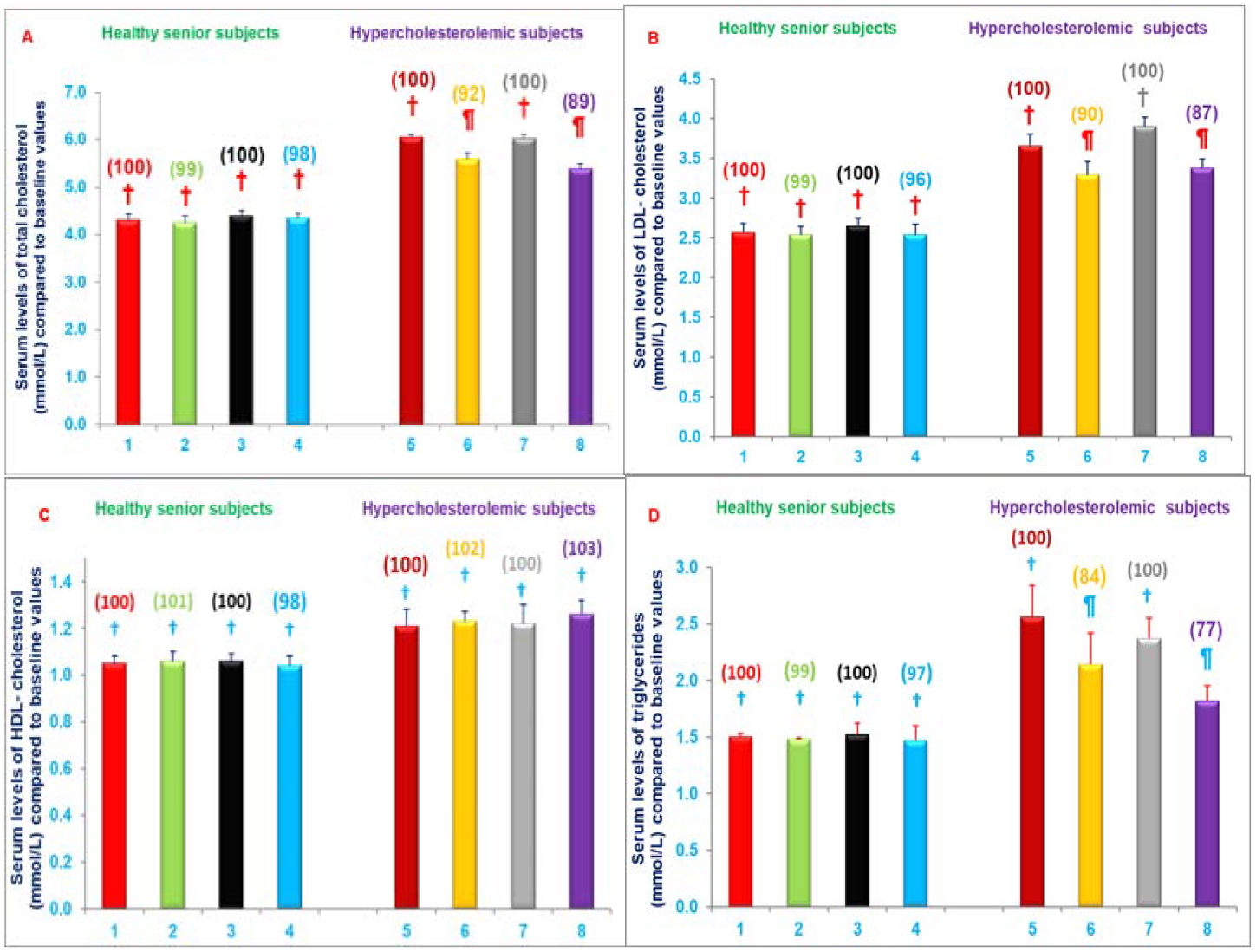
A-D) Impact of a mixture of NS-7 or NS-6 on Serum levels of CRP, NO, TAS or γ-GT in free-living healthy seniors or hypercholesterolemic subjects restricted to AHA Step-1 diet for 4-weeks. Columns 1–4 represent group I-healthy senior subjects. 1. Baseline serum levels of CRP, NO, TAS, or γ-GT (same parameters for all treatments) at the start of NS-7 mixture, 2. After 4-weeks of NS-7 mixture, 3. At the start of NS-6 mixture, 4. after 4-weeks of NS-6 mixture. Similarly, columns 5–8 represent group II-hypercholestrolemic subjects; 5. Baseline at start of NS-7 mixture + AHA Step-1 diet; 6. After 4-weeks of NS-7 mixture + AHA Step-1 diet, 7. At the start of NS-6 mixture + AHA Step-1 diet, 8. After 4-weeks of NS-6 mixture + AHA Step-1 diet. The estimation details of these parameters are in experimental section [29]. Data are means ± SE. Values in a column not sharing a common symbol are significantly different at P<0.05. Percentages of each treatment compared to baseline values are in parenthesis above the column [29].

**Figures 11:**
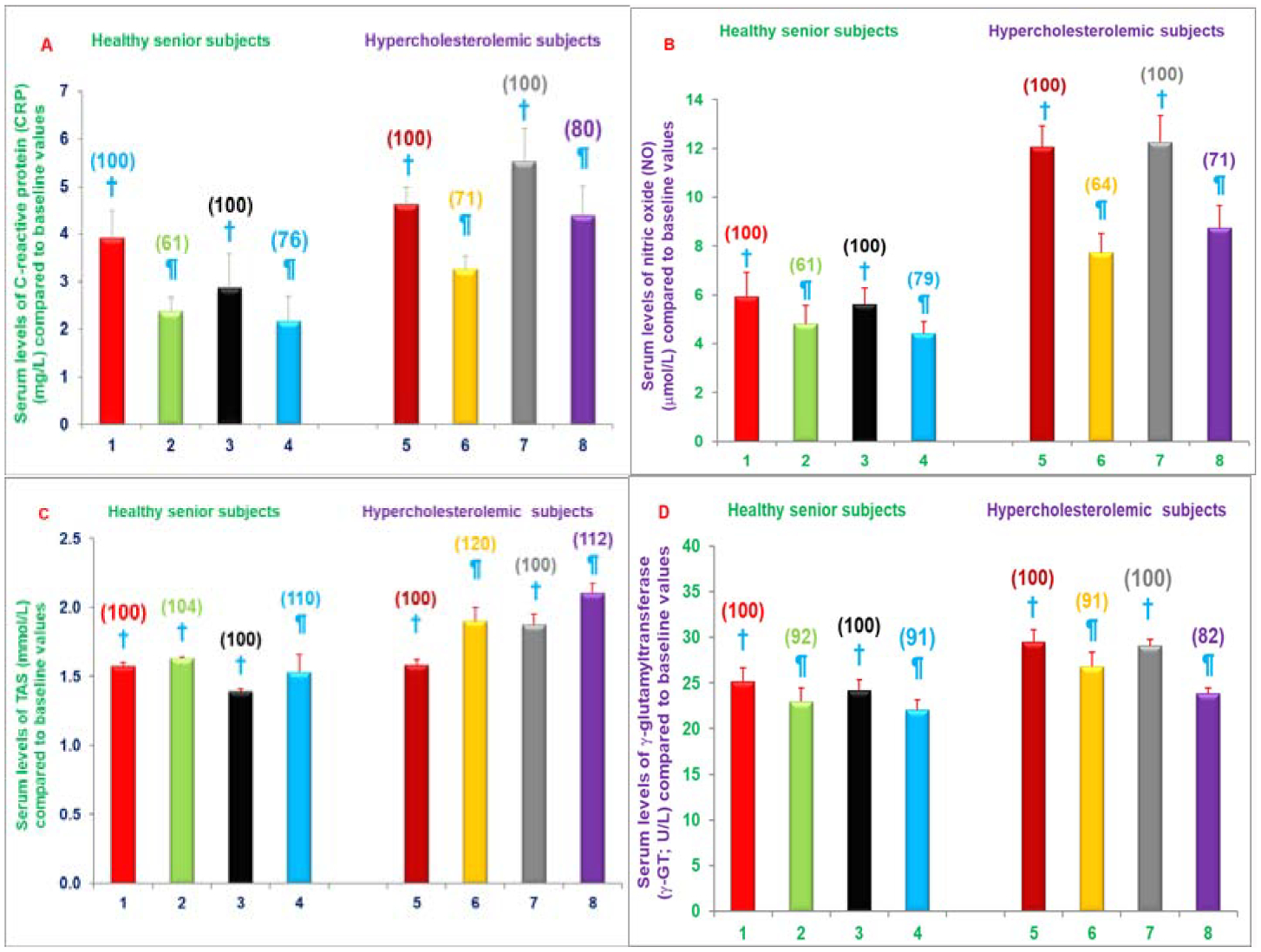
A-D) Impact of administering mixture of NS-7 or NS-6 on Serum levels of lipid parameters in free-living healthy seniors or hypercholesterolemic subjects restricted to AHA Step-1 diet for 4-weeks. Columns 1–4 represent group I- healthy senior subjects. 1. Baseline serum levels of total cholesterol, LDL-cholesterol, HDL-cholesterol, and triglycerides (same parameters for all treatments) at the start of NS-7 mixture, 2. After 4-weeks of NS-7 mixture, 3. At the start of NS-6 mixture, 4. After 4-weeks of NS-6 mixture. Similarly, columns 5–8 represent group II- hypercholestrolemic subjects. 5. Baseline at start of NS-7 mixture + AHA Step-1 diet, 6. After 4-weeks of NS-7 mixture + AHA Step-1 diet, 7. At the start of NS-6 mixture + AHA Step-1 diet, 8. After 4-weeks of NS-6 mixture + AHA Step-1 diet. The estimation details of these parameters are in experimental section [29]. Data are means ± SE. Values in a column not sharing a common symbol are significantly different at P<0.05. Percentages of each treatment compared to baseline values are in parenthesis above the column [29].

The intake of two capsules/d of NS-7 or NS-6 for four weeks significantly decreased serum levels of CRP (19%, 21%), NO (39%, 24%), γ-GT activity (8%, 6%), respectively in free-living healthy seniors and serum levels of CRP (29%, 20%), NO (36%, 29%), γ-GT activity (9%, 18%), total cholesterol (8%, 11%), LDL-cholesterol (10%, 13%), and triglycerides (16%, 23%) levels were decreased in hypercholesterolemic subjects restricted to AHA Step-1, respectively compared to respective control groups (Figures 10A–10D and 11A–11D). The serum levels of TAS were increased (3%, 9%) in free-living healthy seniors and in hypercholesterolemic subjects plus AHA Step-1 diet (20%, 12%) with either of the mixture NS-7 or NS-6 tested (Figures 11A–11D) [29]. Moreover, serum levels of NO were significantly increased in seniors compared to both children (~80%) and young adults (~65%) as shown in Table 6. These results have demonstrated that diet supplementation with resveratrol, pterostilbene, morin hydrate; quercetin, δ-tocotrienol, riboflavin, and nicotinic acid reduce cardiovascular risk factors in humans when used as nutritional supplements with, or without, other dietary changes [29].

Clinical studies using Tocotrienol-Rich Fraction (TRF) from palm oil yielded inconsistent results with regards to its efficacy due to presence of tocopherols in TRF mixture. The impact of tocopherol-free δ-tocotrienol (Figure 1) on inflammatory and oxidative stress biomarkers, plasma cytokines/proteins, their gene expression, and microRNAs was reported in hypercholesterolemic subjects [30]. Hypercholesterolemic (n=31; serum cholesterol >5.2 mmol/L) subjects were enrolled in the study. All hypercholesterolemic subjects were given increasing doses of δ-tocotrienol (125 mg, 250 mg, 500 mg, 750 mg/d) plus AHA Step-1 diet for 4-weeks of each treatment. Serum Nitric Oxide (NO), C-Reactive Protein (CRP), Malondialdehyde (MDA), γ-Glutamyl-Transferase (γ-GT), Total Antioxidant Status (TAS), cytokines/proteins, cDNA, and microRNAs were determined.

**Figures 12:**
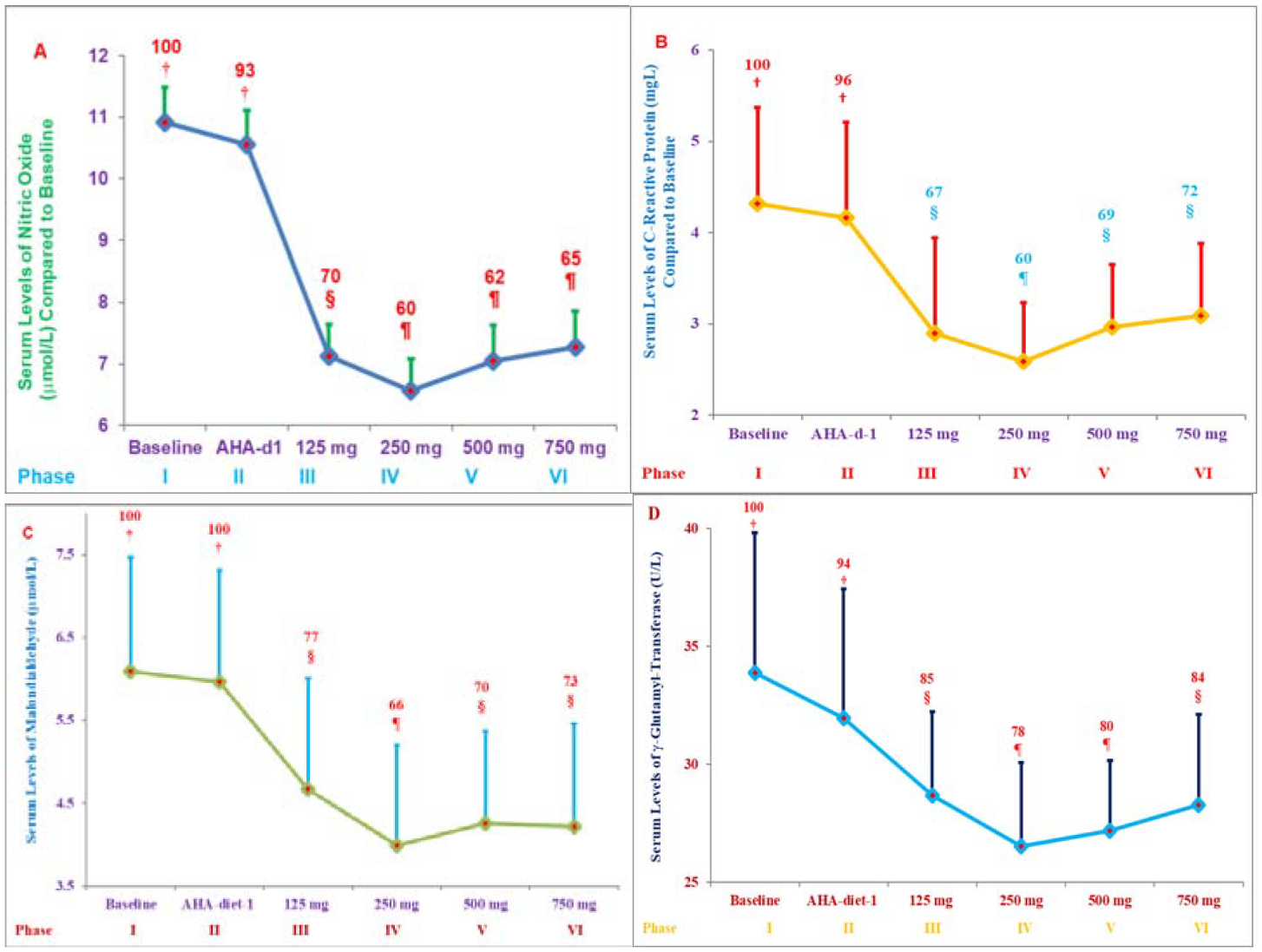
A-E) Role of various doses of δ-tocotrienol plus AHA Step-1 diet on serum levels of nitric oxide (NO; A), C-Reactive Protein (CRP; B), Malondialdehyde (MDA; C), γ-Glytamyl-Transferase (γ-GT; D), and Antioxidant Status (TAS; E) in hypercholesterolemic subjects: The treatments 1–6 correspond to six phases, and each phase lasted for 4 weeks: 1. Baseline (n=31); 2. AHA Step-1 diet; 3. δ-tocotrienol 125 mg/d +AHA Step-1 diet; 4. δ-tocotrienol 250 mg/d +AHA Step-1 diet; 5. δ-tocotrienol 500 mg/d +AHA Step-1 diet; 6. δ-tocotrienol 750 mg/d +AHA Step-1 diet. Data are means ± SD (Standard Deviation). Percentages of each treatment compared to baseline values are above the column. Point on a line not sharing a common symbol are significantly different at §=P<0.01; ¶= P<0.001 [30].

All concentrations of δ-tocotrienol reduced serum levels of NO, CRP, MDA, γ-GT. The most effective dose (250 mg/d) decreased serum NO (40%), CRP (40%), MDA (34%), γ-GT (22%) significantly, while TAS levels increased 22% (Figures 12A–12E) [30].

**Figures 13:**
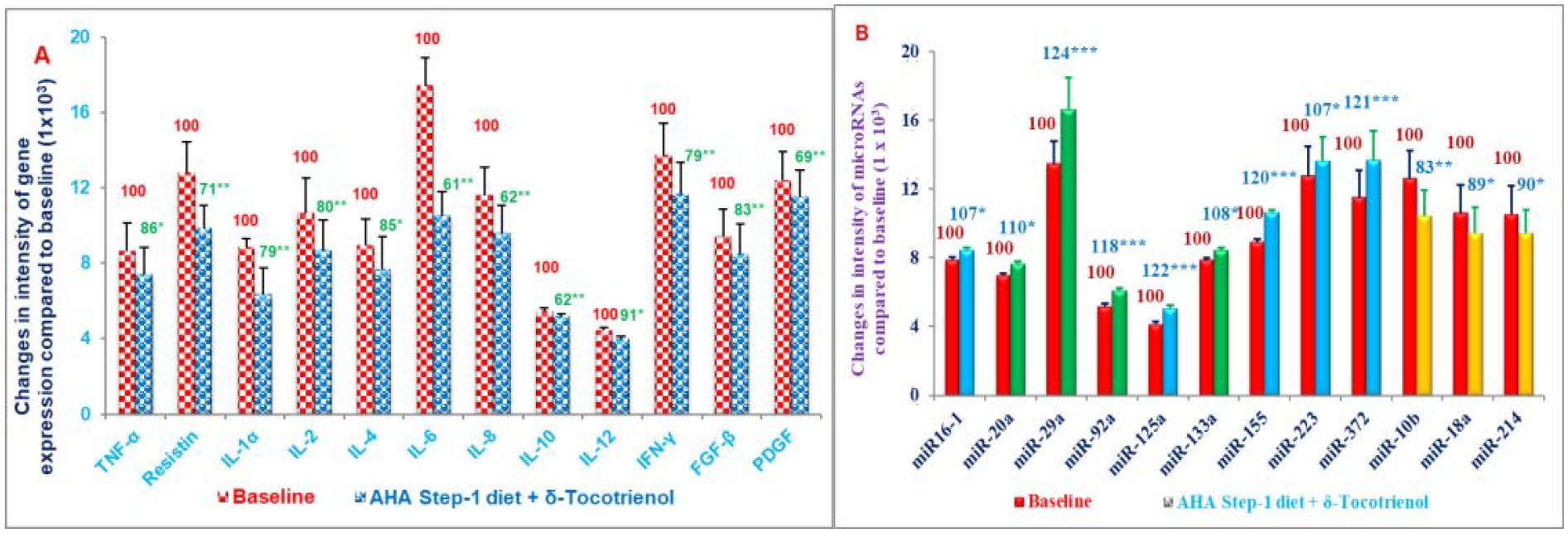
A, B) Effects of δ-tocotrienol (250 mg/d) +AHA Step-1 diet on various plasma gene expression (A), and microRNA (B) in hypercholesterolemic subjects. *-***Number of asterisks indicates differences from respective control values *P<0.05; **P<0.01 (Figure 16A); *-***Represent statistical significance from respective control value, *P<0.05; **P<0.01; ***P<0.001 (Figure 16B) [30].

The 500 mg/d and 750 mg/d doses were less effective in improving oxidative stress compared to the 250 mg/d dose. Inflammatory plasma cytokines (resistin, IL-1α, IL-12, IFN-γ) were reduced 15% to 17%, while cardiac angiogenic Fibroblast Growth Factor-β (FGF-β) and Platelet-Derived Growth Factor (PDGF) were decreased by 11% and 14% with 250 mg/d δ-tocotrienol treatment, respectively (Figure 13A) [30]. Similar results were obtained for cytokine gene expression. Several plasma miRNAs (miRNA-16–1, miRNA-125a, miRNA-133, miRNA-155, miRNA-223, miRNA-372, miRNA-10b, miRNA-18a, miRN-214) associated with cardiovascular disease and cancer were modulated by δ-tocotrienol treatment (Figure 13B). In a dose-dependent study of 125 mg/d to 750 mg/d, δ-tocotrienol maximally reduced inflammation and oxidative stress parameters with a 250 mg/d dose in hypercholesterolemic subjects and may be an attractive therapeutic alternative for the natural maintenance of health during aging process [30].

The inhibitory effects of resveratrol, quercetin, δ-tocotrienol, nicotinic acid on several inflammatory and cardiovascular risk factors has been reported in normal cholesterol and hypercholesterolemic humans (Figure 4). The hypothesis was that combination of cholesterol lowering and inflammatory-reducing properties of mixture of these compounds (NS-5) would be more effective than its individual components in reducing the serum levels of several biomarkers of cardiovascular disease in humans [31]. Moreover, effectiveness of NS-5 mixture and its components has also reported effects on cytokines, gene expression, microRNAs, as this area is gaining importance in the understanding of various transcriptional factors and signal pathways, which regulate several biomarkers in several diseases [31].

Modulation of NS-5 mixture, and its components was reported on superoxide production in HUVEC *in vitro*, and on serum levels of total cholesterol, NO, CRP, TAS, plasma cytokines, gene expression, miRNAs *in vivo* in normal cholesterol and hypercholesterolemic human study, which was carried out as double blind randomized, trial of NS-5 mixture, resveratrol, quercetin, and δ-tocotrienol in free-living healthy and hypercholesterolemic humans. The NS-5 mixture (resveratrol, quercetin, δ-tocotrienol, or nicotinic acid) treatments caused reduction in superoxide production (11% to 24%) in HUVEC [31]. These reductions were more pronounced with LPS-stimulated HUVEC (26% to 40%) compared to pre-dose values [31].

**Figures 14:**
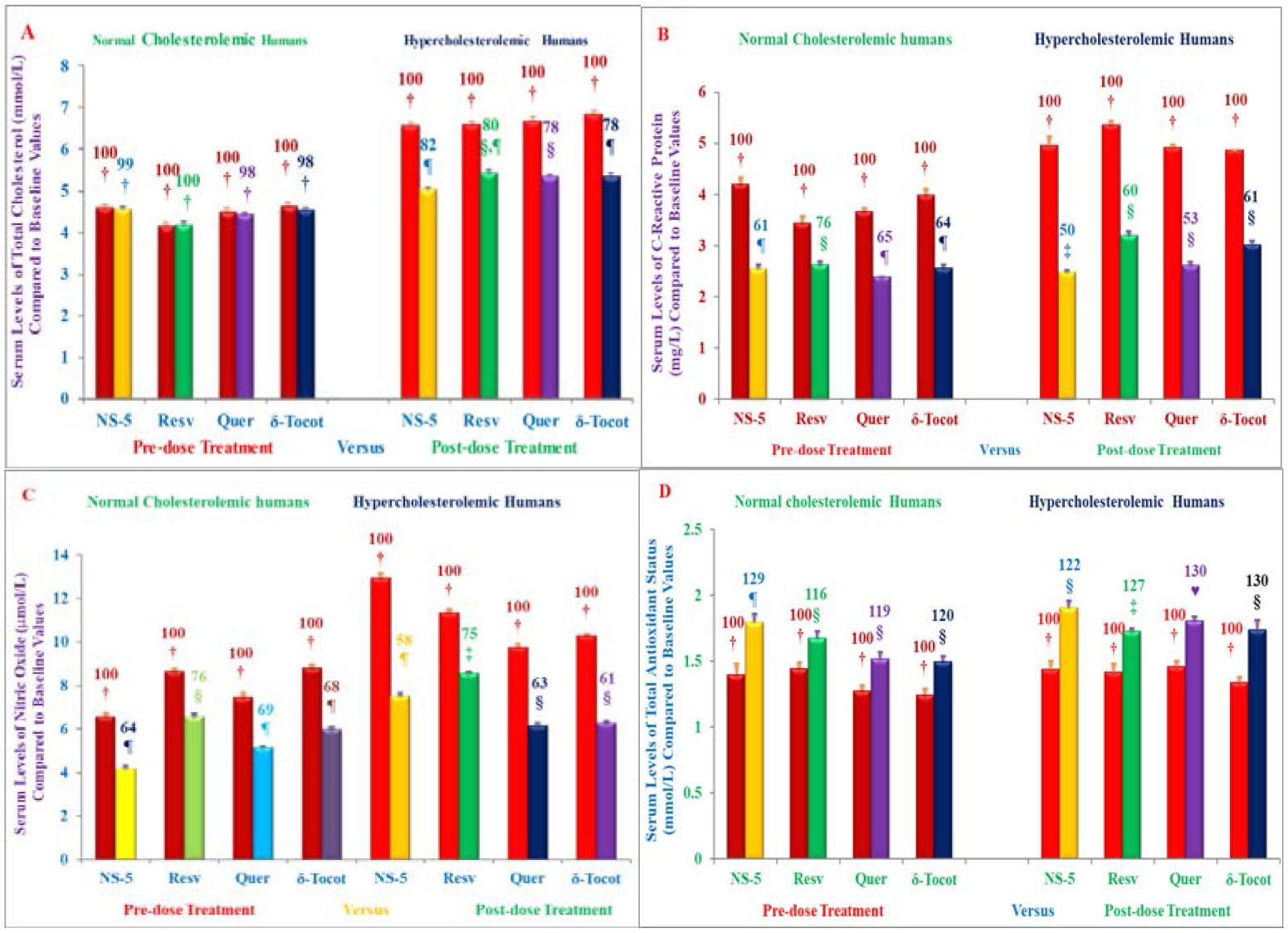
A-D) The Effect of NS-mixture and its components on serum levels total cholesterol, Nitric Oxide (NO) production, C-Reactive Protein (CRP) and Total Antioxidant Status (TAS) in normal cholesterol seniors and hypercholesterolemic subjects plus AHA Step-1 diet. Columns 1–4 represent subjects of group #1 of normal cholesterol humans were administered one capsule of 250 mg/d of NS-5 mixture, or resveratrol, or quercetin, or δ-tocotrienol to normal cholesterol participants for six weeks. The columns 5–8 represent humans of group #2 of hypercholesterolemic participants restricted to AHA Step-1 diet plus one capsule of 250 mg/d of NS-5 mixture or resveratrol, or quercetin or δ-tocotrienol for six weeks. Data are means ± SD (Standard Deviation). Percentages of each treatment compared to pre-dose *vs*. post-dose of each treatment is above the column (Figures 14A–14D). Values in a column not sharing a common symbol are significantly different of various groups at ¶, § P<0.01 [31].

These findings were further supported by decrease in serum total cholesterol levels of NS-5 treated group (24%) *versus* resveratrol (18%), quercetin (20%), and δ-tocotrienol (22%) individually in hypercholesterolemic humans (Figure 14A), followed by reduction of NO, CRP and increases in TAS in normal cholesterol and hypercholesterolemic humans (Figures 14B–14D) [31].

There was significant down-regulation in pro-inflammatory cytokines and gene expressions of resistin, IL-2α, IL-6, IL-12, IL-18, TNF-α, and others, that are normally involved in pathogenesis of atherosclerosis, diabetes, and ageing processes (Table 7, 8). The plasma inflammatory miRNAs (miR-101a, miR-125a, miR-155, miR-223) were down-regulated as compared to pre-dose values. The miRNA-146a increased during senescence and treatment with these compounds down-regulated elevated levels of miRNA-146a (Table 7, 8) [31].

## Conclusions

The anti-inflammatory effects of δ-tocotrienol, quercetin, riboflavin, (−) Corey lactone, amiloride, and dexamethasone on serum TNF-α and NO levels has been reported for the first time. Additionally, all the treatments except with dexamethasone resulted in lower serum total cholesterol, LDL-cholesterol, and triglycerides levels. The mechanism for this inhibition appears to be decreased proteolytic degradation of P-IB protein due to the inhibition of proteasome, resulting in decreased translocation of activated NF-κB to the nucleus, and depressed transcription of gene expression of TNF-α, and *iNOS*. The impact of above compounds was increased on these parameters when combined with δ-tocotrienol.

Moreover, these results also indicated that intravenously administered tocotrienols inhibited acute platelet-mediated thrombus formation, and collagen and ADP-induced platelet aggregation. The δ-tocotrienol, riboflavin, and quercetin inhibit NO production in LPS-stimulated macrophages of all four strains of mice, and TNF-α secretion only in LPS-stimulated macrophages of C57BL/6 and BALB/c mice. Furthermore, these naturally occurring compounds are potent inhibitors of multiple subunits in the proteasomes. Consequently, these agents could potentially suppress the production of inflammatory mediators in ageing humans, thereby decreasing the risk of developing a variety of ageing related diseases.

δ-Tocotrienol and quercetin inhibit the LPS-induced NO production *in vivo*. The microarray RNA analyses, followed by ingenuity pathway analyses indicated that quercetin or δ-tocotrienol inhibit LPS-induced gene expression of several genes involved ageing and pro-inflammation, such as IL-1β, IL-1α, IL-6, TNF-α, IL-12, *iNOS*, VCAM1, ICAM1, COX2, IL-1RA, TRAF1 and CD40. The NF-κB pathway regulates the production of NO and the pro-inflammatory cytokines involved in normal and ageing process. The *ex vivo* results confirmed the earlier *in vitro* findings. Thus, these findings of inhibition of NO production by δ-tocotrienol and quercetin may be of clinical significance treating several inflammatory diseases, including ageing process. This was the first report demonstrating that resveratrol and pterostilbene act as proteasome inhibitors, thus providing a mechanism for their anti-inflammatory effects. These results indicated that serum NO levels are elevated in elderly humans compared to children or young adults. Diet supplementation with combinations of resveratrol, pterostilbene, morin hydrate, quercetin, δ-tocotrienol, riboflavin, and nicotinic acid reduce cardiovascular risk factors in humans when used as nutritional supplements with, or without, other dietary changes.

In a dose-dependent study of 125 mg/day to 750 mg/day, δ-tocotrienol maximally reduced inflammation and oxidative stress parameters with a 250 mg/day dose in hypercholesterolemic subjects and may be an attractive therapeutic alternative for the natural maintenance of health during aging process. The plasma inflammatory miRNAs (miR-101a, miR-125a, miR-155, miR-223) were down-regulated as compared to pre-dose values. The miRNA-146a increased during senescence and treatment with these compounds down-regulated elevated levels of miRNA-146a.

## Figures and Tables

**Table 1:** Microarray analyses of RNA of livers of chickens after treatment with various compounds.

Set-I:			Genes up-regulated					
#	Genes	Control	δ-T3	Quer	Ribo	Co Lac	Dexa	Description
	**Inflammation**							
1	CF256615	1.96	4.37	4.40	3.60	4.55	3.26	Interferon-gamma receptor alpha chain precursor
2	BU313956	2.41	2.75	4.64	2.99	3.24	3.21	Suppressor of cytokine signaling 1
3	AJ720966	2.54	4.72	4.95	4.29	4.66	4.09	Nuclear factor kappaB related to binding protein
4	NM_205485	3.35	4.40	5.91	5.06	5.28	5.10	Interferon 1 receptor, type 1
	**Ageing**							
5	AF175433	1.96	4.00	5.05	3.80	3.50	2.20	T cell receptor delta chain (TCRD)
6	NM_205520	3.23	4.04	5.55	3.66	4.15	3.32	ATPase, Na+/K+ transporting, beta 1 polypeptide
7	AF387865	4.67	6.17	7.02	5.55	6.49	6.44	Heat shock protein 90Da beta (Grp94), member 1
8	BX932093	6.04	8.07	8.34	7.24	8.47	8.01	PIT 54 protein
	**Cardiovascular**							
9	ENSGAL T00000012	2.25	4.26	4.82	3.59	5.05	3.61	Glucose 6-phosphate translocase
10	AJ720577	2.37	4.78	6.86	4.80	6.74	5.97	NADH dehydrogenase (Ubiquinone) Fe-S protein 1
11	NM_204259	2.48	5.88	6.02	5.75	5.70	3.67	Prostaglandin D2 synthase 21KDa
12	AJ720030	3.29	5.00	5.62	5.35	5.13	1.62	Squalene epoxidase
13	NM_205483	3.50	5.60	5.60	4.48	3.97	3.72	Lipoprotein
14	ENSGAL T00000010	3.83	5.08	5.43	5.49	5.89	3.28	Enoyl-coenzyme A, hydratase/3-hydroxyacyl coenz
15	NM_2051155	4.73	5.52	6.87	5.48	5.79	6.09	Fatty acid synthase
16	ENSGAL T00000020	5.09	5.86	7.01	6.32	7.11	5.70	Alcohol dehydrogenase 1C (class 1), γ-polypeptide
17	NM_204605	5.83	7.57	7.98	7.05	7.68	6.68	Coagulation factor II (thrombin)
18	BX931521	6.36	8.27	8.94	8.12	8.43	8.63	Glutathione peroxide 3 precursor (GSHPx-3)
	**Cancer**							
19	AJ720735	0.18	1.76	2.70	0.90	2.87	1.37	Endoplasmic reticulum protein 29
20	BU117693	1.51	2.98	3.56	2.68	2.93	2.48	RAN, member RAS oncogene family
21	ENSGAL T00000006	2.60	5.10	5.88	4.53	5.81	4.98	Similar to cytochrome P450, family 2.
								
Set-II:			Genes down-regulated					
#	Genes	Control	δ-T3	Quer	Ribo	Co Lac	Dexa	Description
	**Inflammation**							
1	AJ719859	3.33	2.03	2.42	1.74	1.93	1.18	Proteasome (prosome, macropain) activator subunit 4
2	BX950555	4.01	4.11	4.38	3.78	3.38	1.64	Tumor necrosis factor superfamily, # 5-induced protein
3	BU397996	4.53	2.82	2.18	2.74	2.20	2.87	Protein Kinase
	**Ageing**							
4	CR353144	4.03	1.66	3.57	3.12	2.92	3.18	Nuclear DNA-binding protein
5	BU426315	4.50	2.20	2.42	2.14	1.70	1.33	Carnitine palmitoyltransferase 1A (liver)
6	CR523285	4.60	2.35	3.26	1.90	3.68	8.02	Finshed cDNA clone ChEST613j16
7	CD735693	6.05	4.25	5.44	4.66	3.68	3.39	Heat shock protein 25
8	BU123548	6.47	6.16	7.31	6.30	6.14	4.20	Finshed cDNA clone ChEST495e19
9	NM_205471	8.73	6.99	8.76	7.69	7.42	6.28	Phosphoenolpyruvate carboxykinase 1
10	CR523582	6.79	5.31	4.78	5.19	4.66	4.84	B-cell CLL/lymphoma 9
	**Cardiovascular**							
11	M64990	3.94	1.69	1.51	2.10	1.02	1.25	Prostaglandin-endoperoxide synthase 2
12	CR522967	4.42	3.41	2.18	3.20	2.53	3.67	KIAA1285 protein
13	ENSGAL T00000024	5.31	3.93	5.00	4.42	4.60	3.05	Aproteindipose differentiation-related
14	BX935098	5.79	4.45	5.41	4.35	4.52	3.09	Glutathione S-transferase theta 1
15	BU422942	5.84	4.14	3.65	4.09	3.79	4.41	Glycogen synthase kinase 3 beta
16	BU272340	6.58	5.63	4.23	5.47	5.39	5.81	Inositol hexaphosphate kinase 2
	**Cancer**							
17	AJ447153	2.19	0.83	2.06	1.56	1.21	0.01	Protein tyrosine phosphate, non-receptor type 2
18	BU463093	3.63	2.67	4.07	2.78	3.28	0.90	Amino acid transporter system A1
19	AL585963	3.95	2.15	1.74	1.20	2.07	1.67	RAS guanyl releasing protein 3
20	ENSGAL T00000000	4.24	3.18	1.62	2.42	1.85	2.97	Breast cancer-associated antigen BRCAA 1
21	BU131710	4.47	4.36	5.24	5.28	4.35	2.31	Isopentenyl-diphosphate delta isomerase 1
22	BU111042	6.54	5.46	4.39	4.81	4.98	5.95	Chrosome 6 open reading frame 111; SR rich protein
23	BU458470	7.84	5.78	7.08	6.96	5.90	5.90	Jun oncogene

δ-T3 = δ-Tocotrienol; Quer. = Quercetin; Ribo = Riboflavin; Co Lac = (−) Corey Lactone; Dexa = Dexamethasone

**Table 2A, B, C:** Effects of α-tocotrienol or α-tocopherol or TRF (5 μg/kg of each per body weight) on the concentration of tocotrienols + tocopherols (tocols) of plasma and platelets of dog^1^.

Treatments	Total Tocopherols (T)^2^	Total Tocotrienols (T3)^3^	Total Tocols (T + T3)
**A. α-Tocotrienol (T3)**	Concentrations in mg/ml (ppm)		
**Plasma (Pre-dose)**	10.10 ± 0.82^a^ (100)^5^	0.90 ± 0.03^a^ (100)	11.02 ± 0.81^a^ (100)
**Plasma (Post-dose)**	45.11 ± 2.19^b^ (447)	2.78 ± 0.13^b^ (309)	47.89 ± 2.17^b^ (435)
			
**Platlets (Pre-dose)**	5.12 ± 1.24^a^ (100)	1.02 ± 0.03^a^ (100)	6.14 ± 1.22^a^ (100)
**Platlets (Post-dose)**	28.16 ± 1.54^b^ (550)	1.28 ± 0.04^b^ (125)	29.44 ± 1.52^b^ (479)
**B. α-Tocopherol (T)**			
**Plasma (Pre-dose)**	17.60 ± 0.32^a^ (100)^5^	2.89 ± 0.05^a^ (100)	20.49 ± 0.26^a^ (100)
**Plasma (Post-dose)**	56.91 ± 0.18^b^ (323)	1.49 ± 0.03^b^ (52)	58.40 ± 1.65^b^ (285)
			
**Platlets (Pre-dose)**	10.05 ± 0.27^a^ (100)	2.84 ± 0.02^a^ (100)	12.89 ± 0.29^a^ (100)
**Platelets (Post-dose)**	42.22 ± 0.21^b^ (420)	2.33 ± 0.04^b^ (82)	44.55 ± 0.22^b^ (346)
**C. Tocotrienol Rich fraction (TRF)** ^4^			
**Plasma (Pre-dose)**	19.72 ± 1.29^a^ (100)^5^	1.65 ± 0.03^a^ (100)	21.37 ± 1.13^a^ (100)^6^
**Plasma (Post-dose)**	67.38 ± 2.25^b^ (342)	8.56 ± 0.04^b^ (519)	75.94 ± 2.21^b^ (355)
			
**Platlets (Pre-dose)**	7.85 ± 0.62^a^ (100)	3.85 ± 0.23^a^ (100)	11.70 ± 0.48^a^ (100)
**Platelets (Post-dose)**	31.44 ± 1.84^b^ (401)	5.84 ± 0.12^b^ (152)	35.28 ± 1.70^b^ (302)

1 Data expressed as means + SD = 3, 3, and 4 respectively per treatment.

2 Total tocotrienols = Mixture of α-tocotrienol + β-tocotrienol + γ-tocotrienol + δ-tocotrienol (T3).

3 Total tocopherols = Mixture of α-tocopherol + β-tocopherol + γ-tocopherol + δ-topherol (T)

4 TRF = Tocotrienol rich fraction (Mixture of α-tocotrienol 15% + γ-tocotrienol 60% + δ-tocotrienol 25%).

5 Percentsge of control values are in parenthesis.

a-b Values in columns with a different superscript letter are significantly different at P < 0.05.

**Table 3A:** Effects of various compounds on weight gain of 8-week-old and 46-week-old C57BL/6 male mice after feeding for 4 weeks^1^.

NO		Weight gain in gm (8-week-old)			Weight gain in gm (46-week-old)	
	C57BL/6 male mice^2^	Pre-Feeding	Post-Feeding		Pre-Feeding	Post-Feeding
1	Control Diet (CD)	21.43 ± 2.25^a^ (100)^3^	27.54 ± 2.56^b^ (129)^3^		30.32 ± 2.63^a^ (100)^3^	37.31 ± 3.46^b^ (123)^3^
2	CD + Quercetin (100 ppm)	22.72 ± 2.42^a^ (100)	27.92 ± 2.37^b^ (123)		28.47 ± 2.52^a^ (100)	36.62 ± 3.76^b^ (129)
3	CDA + δ-Tocotrienol (100 ppm)	20.25 ± 2.26^a^ (100)	26.42 ± 2.51^b^ (130)		30.33 ± 2.64^a^ (100)	37.32 ± 3.38^b^ (123)
4	CD + Dexamathasone (10 ppm)	21.47 ± 2.62^a^ (100)	23.53 ± 2.41^a^ (110)		31.58 ± 3.22^a^ (100)	33.61 ± 3.21^a^ (106)

1 Each compound (200 μg or 20 μg) was dissolved in ethanol (100 μL) and mixed with powder commercial diet (2 kg).

2 Data are means ± SD, n = 3 mice per group.

3 Percentage of gain in weight in each group compared to respective control group are in parenthesis.

a-b Values in a row not sharing a common superscript letter are significantly different at P < 0.05.

**Table 3B:** Effects of various compounds on the gene expression of TNF-α and iNOS in LPS-stimulated thioglycolate-slicited peritoneal macrophages derived from 8-week-old and 46-week-old C57BL/6 male mice after feeding for 4-weeks^1^.

8-week-old vs 46-week-old		RT-PCR (8-week-old) data^2^			RT-PCR (46-week-old) data^2^	
NO		Gene Expression			Gene Expression	
	C57BL/6 Male Mice	TNF-α	iNOS		TNF-α	iNOS
1	Media + Macrophages (MM)	0	0		0	0
2	MM + LPS, 10.0 *ng*/mL (A)	0.35 (100)*^3^	0.41 (100)^3^		0.38 (100)^3^	0.69 (100)^3^
3	A + Quercetin (100 ppm)	0.26 (74)	0.29 (71)		0.29 (76)	0.47 (68)
4	A + δ-Tocotrienol (100 ppm)	0.24 (69)	0.22 (54)		0.20 (53)	0.28 (41)
5	A + Dexamethasone (10 ppm)	0.20 (57)	0.15 (37)		0.27 (71)	0.20 (29)

	*Control groups	8-week-old vs	46-week-old
1	**TNF-α**	**0.35 (100)**	**0.38 (109)**
2	**iNOS**	**0.41 (100)**	**0.69 (168)**

1 The assay mixture (A; contains 0.2% dimethyl sulfoxide = DMSO) was treated with LPS (10.0 ng/well) for 4 h.

2 Total RNA was extracted using RNAeasy mini Kit.

3 Percentage of control ratio, based on MM + LPS 10.0 ng/assay.

**Table 4:** Inhibitory effects of various compounds (concentrations of 10 – 320 μM) on chymotrypsin-like proteasomal activity in RAW 264.7 cells^1^.

NO	Assay mixture	Resveratrol	Pterostilbene	Morin Hydrate	Nicotinic acid	Quercetin
			Average digital values of relative luminescence units (RLU)			
1	Medium + cells = A	87458	87458	87458	87458	87458
2	A + DMSO control^2^	95222 ± 2014 (100)^3^	95222 ± 2014(100)^3^	95750 ± 2014 (100)^3^	95750 ± 2014 (100)^3^	95750 ± 2014 (100)^3^
3	10 μM	7376 ± 107 (8)	27543 ± 947 (29)	89557 ± 1958 (94)	95240 ± 2020 (99)	61823 ± 2206 (65)
4	20 μM	6996 ± 143 (7)	24177 ± 1775 (25)	67338 ± 1626 (71)	95342 ± 1896 (100)	36772 ± 1201 (39)
5	40 μM	2742 ± 138 (3)	23574 ± 1126 (25)	55078 ± 1444 (58)	94232 ± 1962 (98)	19805 ± 732 (21)
6	80 μM	2261 ± 124 (2)	21639 ± 1667 (23)	41178 ± 1099 (43)	94155 ± 1926 (98)	17478 ± 783 (18)
7	160 μM	1688 ± 121 (2)	19483 ± 1207 (20)	33814 ± 134 (36)	95165 ± 1966 (99)	13827 ± 621 (15)
8	320 μM	1590 ± 92 (2)	13380 ± 1341 (14)	27448 ± 88 (29)	94124 ± 2038 (98)	5896 ± 321 (6)

1 Proteasome-Glo (Promega) chymotrypsin-like cell-based assay was used. The chymotrypsin-like acitivity was quantitated by measuring luminescence after treating of RAW 264.7 whole cells (1 ×10^4^) with various concentrations (10 μM – 320 μM) of resveratrol, pterostilbene, morin hydrate, nicotinic acid, or quercetin as described in Methods. The relative luminescence units (RLU) of assays were read in Promega Plate Luminoter.

2 Medium + RAW 264.7 whole cells + 0.2% dimethyl sulfioxide (DMSO) was used as control.

3 Percentages based on digital values of RLU compared to control are in parenthesis.

**Table 5:** Inhibition of various compounds on the gene expression of TNF-α, IL-1β, IL-6, and iNOS in LPS-stimulated RAW 264.7 cells^1^.

NO	Treatments	RT-PCR data (Ratios of digital values of optical density of gene expression of cytokine/b-actin).			
		TNF-α	IL-1β	IL-6	iNOS
1	Media + Cells = A	0.05	0.05	0.10	0.20
2	A + LPS (10 ng/well) = B	0.83	1.35	0.93	1.12
3	B + 0.2% DMSO = C	0.80 ± 0.03^a^ (100)^2^	1.32 ± 0.03^a^ (100)^2^	0.96 ± 0.03^a^ (100)^2^	1.04 ± 0.02^a^ (100)^2^
4	C + Resveratrol (16.0 μM)	0.18 ± 0.02^e^ (23)	0.51 ± 0.03^e^ (39)	0.21 ± 0.01^e^ (22)	0.65 ± 0.02^c^ (63)
5	C + Pterostilbene (16.0 μM)	0.38 ± 0.03^d^ (48)	0.75 ± 0.03^d^ (57)	0.62 ± 0.03^c^ (65)	0.92 ± 0.03^b^ (88)
6	C + Morin hydrate (16.0 μM)	0.55 ± 0.03^b^ (69)	0.92 ± 0.04^c^ (70)	0.63 ± 0.02^c^ (66)	0.53 ± 0.03^d^ (51)
7	C + Nicotinic acid (16.0 μM)	0.76 ± 0.05^a^ (95)	1.12 ± 0.02^b^ (85)	0.75 ± 0.03^b^ (78)	0.66 ± 0.05^c^ (64)
8	C + Quercetin (16.0 μM)	0.45 ± 0.02^c^ (56)	0.22 ± 0.02^f^ (17)	0.25 ± 0.01^d^ (26)	0.37 ± 0.03^e^ (36)

1 The RAW 264.7 cells (1×10^7^ cells/well) were adhered to 12 well plate for 4 h. After 4h, the cells were washed, cultured overnight in fresh medium (500 μL) at 37°C. Then cells were treated with 16 μM of various compounds for 1 h. After 1 h, LPS (10 ng/well) was added in each well. The cells were harvested after 4 h incubation at 37°C to extract total RNA with RNeasy mini kit as described in methods. The total RNA of each treatment was transcribed and analyzed by real-time polymerase chain reaction (RT-PCR) to quantitate gene expression of TNF-α, IL-1β, IL-6, and iNOS. Cell viability was >95% in all the treatments. Data are means ± SD, n = 3 (triplicate analysis of each sample).

2 Percentages of digital values of relative optical density of each cytokine/b-actin of each treatment are in parenthesis.

a-f Values in a column not sharing a common superscript letter are significantly different at P < 0.05.

**Table 6:** The plasma levels of nitric oxide (NO) of free-living healthy human subjects of various ages^1^.

#	Parameters	Boys	Girls		Adult males	Adult females		Senior males	Senior females
1	Age (Years)	3.25 ± .25**	3.19 ± 0.25		32.81 ± 1.11	31.13 ± 0.70		68.63 ± 0.93	67.25 ± 0.40
2	Number of subjects (*n*)	16	16		16	16		16	16
3	*Plasma levels of nitric oxide in μM	11.36 ± 0.77**	13.20 ± 0.58		12.43 ± 0.70	14.31 ± 0.44		20.38 ± 0.91	24.03 ± 1.56
								179.40%***	182.05%***
		**12.28 (μM)**						**22.21 (μM; 180.86%)**	

1 The estimations of plasma nitric oxide (NO) were carried out according to published procedure as described in Materials and Methods.

* Average of triplicalte analyses of each sample.

*** Compared to respective values of baby boys and baby girls.

**Table 7:** Effects of NS-5, resveratrol, quercetin, & δ-tocotrienol (Pre-dose vs Post-dose) on gene expression of messenger RNAs (RNAs) of normal choles-terolemic humans.

			NS-5	Resveratrol	Quercetin	δ-Tocotrienol	
#	Genes^¶^	Pre-dose	Post-dose	Post-dose	Post-dose	Post-dose^‡^	Descriptions
			%	%	%	%	
**I**	**Cardiovascular:**						
1	Resistin	100	48 ± 3.22**	76 ± 1.12**	57 ± 1.35**	73 ± 2.81**	Pathogenesis of obesity-mediated insulin
							resistance and type 2 diabetes mellitus
2	IL-2	100	53 ± 2.07**	88 ± 2.48*	64 ± 2.59**	72 ± 2.88**	Interleukin-2
3	IL-6	100	56 ± 3.06**	71 ± 0.68**	85 ± 0.91**	68 ± 2.87*	Interleukin-6
4	IL-8	100	68 ± 1.22**	94 ± 2.90*	95 ± 0.76*	95 ± 0.21*	Interleukin-8
5	IL-12	100	85 ± 1.93**	78 ± 1.51**	70 ± 2.12**	81 ± 0.89**	Interleukin-12
6	1L-17α	100	66 ± 3.44**	83 ± 1.56**	83 ± 0.46**	85 ± 1.62**	Insulin-enhances nitric oxide, & NF-κB.
7	IL-18	100	45 ± 3.08**	59 ± 1.30**	85 ± 1.34**	79 ± 3.00**	Interleukin-18
8	COX-2	100	41 ± 2.78**	73 ± 1.44**	55 ± 1.36**	65 ± 1.62**	Cyclooxygenase-2
9	GAPDH	100	29 ± 2.59**	81 ± 1.36**	73 ± 0.88**	78 ± 2.96**	Glyceraldehyde-3-phosphate dehydrogenase
10	IP-10	100	21 ± 2.15**	88 ± 1.97*	74 ± 1.74**	72 ± 0.94**	Interferon-Inducible Protein-10
							
**II**	**Inflammation:**						
11	TNF-α	100	48 ± 2.30**	63 ± 1.31**	65 ± 2.44**	83 ± 2.08**	Tumor Necrosis Factor-α
12	INF-γ	100	59 ± 2.36**	67 ± 1.10**	71 ± 1.31**	75 ± 1.60**	Interferon-γ
13	MIP-1α	100	57 ± 3.94**	87 ± 1.13*	81 ± 3.20**	74 ± 2.24**	Macrophage Inflammatory Protein-1α
14	NOS-2	100	56 ± 0.41**	57 ± 1.94**	67 ± 1.72**	67 ± 2.11**	Nitric oxide synthase-2
15	VCAM-1	100	70 ± 2.01**	90 ± 3.76*	72 ± 2.93**	84 ± 0.70**	Vascular cell adhesion molecule
16	MCP-1	100	69 ± 4.99**	83 ± 2.83**	77 ± 2.17**	76 ± 2.02**	Monocyte Chemotactic Protein-1
							
**III**	**Cancer:**						
17	IGF-1	100	68 ± 3.56**	74 ± 2.33**	70 ± 2.99**	71 ± 2.93**	Insulin-like Growth Factor-1
18	P-53	100	217 ± 4.87**	113 ± 1.19*	120± 1.96**	239 ± 8.66**	P-53 tumor suppressor protein
19	FAS-1	100	157± 3.72**	119 ± 2.78**	151 ± 3.07**	129 ± 1.94**	Fatty acid synthetase-1
20	VEGF	100	71 ± 2.51**	73 ± 1.78**	83 ± 0.85**	90 ± 0.47*	Vascular Endothelial Growth Factor
21	CCND-1	100	40 ± 5.33**	71 ± 6.04**	54 ± 1.91**	67 ± 3.71**	Cyclin D-1
							
**IV**	**Diabetes:**						
22	PAI-1	100	26 ± 1.21**	81 ± 0.48**	71 ± 4.79**	71 ± 2.37**	Plasmenogen Activator Inhibitor-1
							
**V**	**Aging:**						
23	IL-1α	100	70 ± 1.67**	84 ± 1.52*	84 ± 2.09*	80 ± 0.76**	Interleukin-1α
24	IL-10	100	76 ± 1.44**	96 ± 1.58*	88 ± 4.12*	69 ± 1.42**	Interleukin-10

* - ** Values in a row sharing a common asterisk are significantly different at *P < 0.001; **P < 0.001.

¶ The effect of d-tocotrienol on resistin, IL-2, IL-6, IL-8, IL-10, IL-12, TNF-α & IFN-γ has been reported (AAQ et al. BJMMR. 2015; 6(4): 351–366).

‡ The cytokines IL-12, IL-17α, IL-18 (inflammation); IL-2 (cancer); & resistin, IL-6, IL-17α (diabetes) also involved in these diseases.

**Table 8:** Effects of NS-5, resveratrol, quercetin, & δ-tocotrienol (Pre-dose vs Post-dose) on gene expression of messengerRNAs (mRNAs) of hypercholesterolemic humans.

			NS-5	Resveratrol	Quercetin	δ-Tocotrienol	
#	Genes^¶^	Pre-dose	Post-dose	Post-dose	Post-dose	Post-dose^‡^	Descriptions
			%	%	%	%	
**I**	**Cardiovascular:**						
1	Resistin	100	39 ± 3.02**	43 ± 1.21**	52 ± 1.96**	72 ± 1.04**	Pathogenesis of obesity-mediated insulin
							resistanse & type 2 diabetes mellitus.
2	IL-2	100	28 ± 1.99**	73 ± 1.52**	79± 2.64**	71± 1.71**	Interleukin-2.
3	IL-6	100	56 ± 2.20**	76 ± 2.02**	61 ± 0.89**	62 ± 1.36**	Interleukin-6.
4	IL-8	100	65 ± 1.22**	69 ± 1.22**	77 ± 2.62**	67 ± 2.36**	Interleukin-8.
5	IL-12	100	18 ± 0.62**	78 ± 1.03**	70 ± 2.06**	71 ± 3.40**	Interleukin-12.
6	1L-17α	100	63 ± 2.17**	82 ± 1.86**	69 ± 0.89**	84 ± 0.54**	Insulin-enhances nitric oxide & NF-kB.
7	IL-18	100	75 ± 1.24**	59 ± 1.30**	85 ± 1.34**	79 ± 3.00**	Interleukin-18.
8	COX-2	100	69 ± 1.35**	69 ± 0.83**	61 ± 2.68**	70 ± 1.52**	Cyclooxygenase-2.
9	GAPDH	100	82 ± 1.76**	88 ± 2.03*	88 ± 1.00*	71 ± 2.66**	Glyceraldehyde-3-Phosphate Dehydrogenase.
10	IP-10	100	83± 1.61**	37 ± 1.85**	30 ± 8.50**	19 ± 2.64**	Interferon-Inducible Protein-10.
**II**	**Inflammation:**						
11	TNF-α	100	52 ± 1.80**	57 ± 2.11**	27 ± 0.87**	87 ± 0.94*	Tumor Necrosis Factor-α.
12	INF-γ	100	61 ± 2.57**	73 ± 2.48**	45 ± 3.0**	64 ± 1.90**	Interferon-γ.
13	MIP-1α	100	62 ± 1.95**	73 ± 1.55**	67 ± 0.46**	64 ± 1.53**	Macrophage Inflammatory Protein-1α.
14	NOS-2	100	61 ± 2.02**	62 ± 1.62**	76 ± 0.81**	64 ± 4.24**	Nitric oxide synthase-2.
15	VCAM-1	100	83 ± 2.36**	83 ± 2.59**	72 ± 0.72**	84 ± 0.91**	Vascular Cell Adhesion Molecule.
16	MCP-1	100	35 ± 2.44**	60 ± 3.22**	36 ± 2.83**	39 ± 0.66**	Monocyte Chemotactic Protein-1.
**III**	**Cancer:**						
17	IGF-1	100	64 ± 4.03**	74 ± 1.99**	65 ± 1.49**	61 ± 3.69**	Insulin-like Growth Factor-1.
18	P-53	100	165 ± 2.43**	159 ± 3.27**	126 ± 0.57**	197 ± 2.96**	P-53 tumor suppressor protein.
19	FAS-1	100	155 ± 3.65**	148 ± 1.44**	129± 1.41**	132 ± 0.89**	Fatty Acid Synthetase-1.
20	VEGF	100	65 ± 2.09**	73 ± 3.84**	71 ± 0.93**	78 ± 1.74**	Vascular Endothelial Growth Factor.
21	CCND-1	100	27 ± 4.89**	48 ± 1.37**	71 ± 0.59**	63 ± 3.31**	Cyclin D-1.
							
**IV**	**Diabetes:**						
22	PAI-1	100	33 ± 1.61**	76 ± 1.15**	57 ± 2.93**	72 ± 1.02**	Plasminogen Activator Inhibitor-1.
							
**V**	**Aging:**						
23	IL-1α	100	45 ± 3.15**	72 ± 1.84**	53 ± 1.99**	66 ± 2.76**	Interleukin-1α.
24	IL-10	100	17 ± 1.91**	45 ± 1.79**	57 ± 2.66**	45 ± 0.66**	Interleukin-10.

* - ** Values in a row sharing a common asterisk are significantly different at *P < 0.001; **P < 0.001.

¶ The effect of d-tocotrienol on resistin, IL-2, IL-6, IL-8, IL-10, IL-12, TNF-α & IFN-γ has been reported (AAQ et al. BJMMR. 2015; 6(4): 351–366).

‡ The cytokines IL-12, IL-17α, IL-18 (inflammation); IL-2 (cancer); & resistin, IL-6, IL-17α (diabetes) also involved in these diseases.

## Abbreviations

T2DM: Type 2 Diabetes Mellitus
CRP: C-Reactive Protein
γ-GT: γ-Glutamyltransferase
LPS: Lipopolysaccharide
TNF-α: Tumor Necrosis Factor-α
NO: Nitric Oxide
NF-κB: Nuclear Factor-kappaB
IκB: Interferon kappaB
*iNOS*: Inducible Nitric Oxide
IL-4: Interleukin-4
IL-6: Interleukin-6
